# Polymeric Networks Containing Amine Derivatives as Organocatalysts for Knoevenagel Reaction within Continuously Driven Microfluidic Reactors

**DOI:** 10.3390/gels9030171

**Published:** 2023-02-21

**Authors:** Naresh Killi, Julian Bartenbach, Dirk Kuckling

**Affiliations:** Department of Chemistry, Faculty of Science, Paderborn University, Warburger Str. 100, 33098 Paderborn, Germany

**Keywords:** Knoevenagel reaction, organocatalysis, polymeric gel dots, microfluidic reactions, polymeric networks

## Abstract

The Knoevenagel reaction is a classic reaction in organic chemistry for the formation of C-C bonds. In this study, various catalytic monomers for Knoevenagel reactions were synthesized and polymerized via photolithography to form polymeric gel dots with a composition of 90% catalyst, 9% gelling agent and 1% crosslinker. Furthermore, these gel dots were inserted into a microfluidic reactor (MFR) and the conversion of the reaction using gel dots as catalysts in the MFR for 8 h at room temperature was studied. The gel dots containing primary amines showed a better conversion of about 83–90% with aliphatic aldehyde and 86–100% with aromatic aldehyde, compared to the tertiary amines (52–59% with aliphatic aldehyde and 77–93% with aromatic aldehydes) which resembles the reactivity of the amines. Moreover, the addition of polar solvent (water) in the reaction mixture and the swelling properties of the gel dots by altering the polymer backbone showed a significant enhancement in the conversion of the reaction, due to the increased accessibility of the catalytic sites in the polymeric network. These results suggested the primary-amine-based catalysts facilitate better conversion compared to tertiary amines and the reaction solvent had a significant influence on organocatalysis to improve the efficiency of MFR.

## 1. Introduction

In the past two decades, amine-based catalysis has gained significant attention in the field of organocatalysis to perform various organic reactions [1,2,3,4]. Amines are a class of organic compounds that contain nitrogen atoms and they are classified as primary, secondary and tertiary amines based on the number of alkyl groups present on the nitrogen atom. These amines have been used as catalysts for the transformation of carbonyl groups due to their ability to form iminium ions and enamines as intermediates. These intermediates can be involved in the rate-determining step of the reaction and can also influence the stereo-selectivity of the reaction. The formation and decomposition of these intermediates can be reversible and the direction of the reaction can be influenced by the relative stability of the intermediates and transition states [5]. Moreover, these amines are used as a base for the deprotonation of active hydrogen. However, the reactivity of the amines facilitates the efficiency of the catalyst and the conversion of the reaction. Consequently, comparative studies of catalysis with various amines have a very good scope for performing organocatalysis.

**Figure 1:**
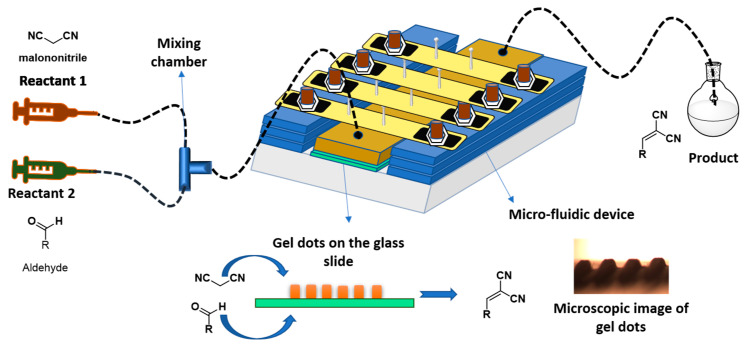
Schematic design of microfluidic system (dimensions of the microfluidic device are width 9 cm, length 10 cm and height 3 cm).

The Knoevenagel reaction is a named reaction in organic chemistry for the formation of C–C bonds, including the synthesis of various biological active molecules [6]. Here, aldehydes react with the active methylene group of strongly activated carbonyl compounds in the presence of amine as a catalyst [7]. Furthermore, most of the amine-based catalytic reactions are performed in homogenous state. However, heterogeneous catalysis is widely used in industrial applications because of the easy separation of the catalyst and lower cost of the operation method [8]. Hence, there is a need for the development of a heterogeneous amine-based catalyst. In previous studies, the Knoevenagel reaction was conducted in the presence of tertiary amine as a heterogeneous catalyst in a continuous flow, which showed better conversion compared to batch reactions [9]. Furthermore, flow chemistry is one of the more advanced technologies in the field of modern chemistry to execute continuous organic reactions and is used in various fields, especially in medicinal chemistry such as drug discovery, organ catalysis, protein separation, etc. [10,11,12,13]. In this continuous reaction process, the reactants are passed through the narrow channels of a fixed catalyst at a controlled flow rate and the product is collected after the successful reaction. In addition, continuous flow reactions are more attractive for organocatalysis because during the catalytic reaction, the product is separated from the catalyst. Moreover, microfluidic reactors (MFR) systems show better sensitivity, lower scale reactions, a green chemistry approach and high conversion of the reaction, compared to the batch reactions [14,15]. The MFR setup contains various components, mainly syringe pump, MFR, tubing system and collector, as shown in Figure 1.

The reaction was carried out in the MFR. In that, the microfluidic chip is the active part for catalysis. Hence, developing a new microfluidic chip is one of the challenges in the preparation of the microfluidic device. Various microfluidic chips have been developed using different materials which can be inorganic and organic [16]. However, organic materials such as polymeric networks have unique properties such as high porosity, swelling in the reaction environment, good mechanical properties, homogeneous distribution of the catalyst and high loading efficiency of the catalysts [17,18,19]. In addition, the swelling properties of the polymeric networks play a significant role in the organocatalysis when the polymer is used as a three-dimensional scaffold. During the organocatalytic reactions, the polymeric networks are swollen in the reaction mixture and the accessibility of the catalytic sites are increased with the increase in swelling [20]. Subsequently, an improved conversion of the reaction and high turnover frequency of the catalyst can be obtained. Similarly, various polymeric networks have been developed in the form of micro or macrogels with the loading of various catalysts to perform several organic reactions, such as the aldol reaction [21], dimerization, Knoevenagel reaction, Baylis–Hillman reaction, Mannich reaction, etc., in the batch method [22,23,24]. Furthermore, packed-bed flow reactors are a type of reactor that uses a dense assembly of a catalytically active polymeric material as a carrier to facilitate chemical reactions. They require high pressure, the high loading of catalysts and a fixed, non-active volume in flow reactor [25,26]. However, low-density crosslinked polymer networks are mechanically rather weak; hence, the polymeric networks’ structure collapses at high pressure during the flow reaction. To overcome the above limitations, micro-structures of polymeric gel dots via photolithography facilitate the stabilization of polymeric networks with a high conversion rate during the microfluidic reactions. In addition, this technology is used for the immobilization of different active molecules (catalyst) when conducting various reactions [9,21,27]. Hence, the synthesis of various amine-based catalysts was performed, and various compositions of monomeric solution were polymerized via photolithography in the form of gel dots as catalysts. Those gel dots were used for continuous flow reactions in MFR, and the catalytic activity of various gel dots for the Knoevenagel reaction, using different aldehydes with malononitrile, was studied. Furthermore, the swelling behavior of the gels and influence of the reaction solvent on the conversion of the reaction at room temperature were evaluated.

## 2. Results and Discussion

**Figure 2:**
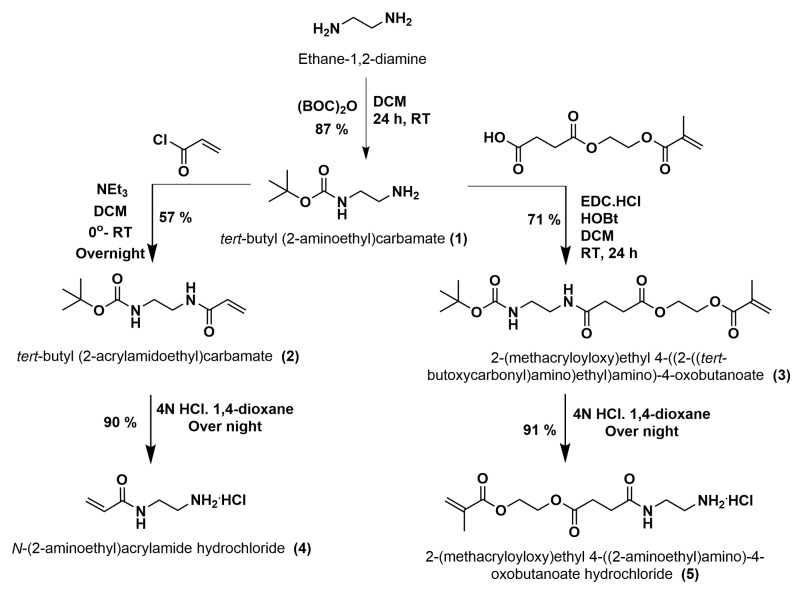
Scheme for the synthesis of catalytic monomers **4** and **5**.

In this research work, we have evaluated the Knoevenagel reaction between different aldehydes and malononitrile (MN) in MFR with polymeric dots, using different amines immobilized as catalysts. Comparative studies of the various amine-based gel dots in the microfluidic systems facilitate the understanding of favorable conditions for the organocatalysis of the Knoevenagel reaction to synthesize different organic entities. In this context, the synthesis of primary-amine-based catalytic monomers, **4** and **5**, were completed in three steps as shown in Figure 2. In the first step, ethylenediamine was used as a precursor material for the synthesis of a mono derivative of BOC-protected ethylenediamine, *tert*-butyl *N*-(2-aminoethyl)carbamate (**1**), as an intermediate compound. Furthermore, acrylate derivatives, *tert*-butyl *N*-[2-(prop-2-enoylamino)ethyl]carbamate (**2**) and 2-(methacryloyloxy)ethyl-4-((2-((*tert*-butoxycarbonyl)amino)ethyl)amino)-4-oxobutanoates (**3**) were synthesized by the reaction between compound **1** and the acrylate monomers, acryloyl chloride and *mono*-2-(methacryloyloxy)ethyl succinate, respectively. The obtained acrylate derivative **2** was purified by crystallization from toluene to obtain **2** as colorless crystals. Furthermore, **3** was purified by column chromatography using 10% of ethyl acetate in *n*-hexane (RF value 0.72) to obtain **3** as a colorless solid. Finally, the deprotection of these acrylate derivatives, **2** and **3**, was carried out using 4N HCl in 1,4-dioxane to yield HCl salts of the primary-amine-based catalytic monomers, *N*-(2-aminoethyl)prop-2-enamide hydrochloride (**4**) and 2-(methacryloyloxy)ethyl-4-((2-aminoethyl)amino)-4-oxobutanoate hydrochloride (**5**). The synthesized primary amine **5** had a longer chain length and was more flexible compared to the primary amine, **4**. Hence, these two different catalytic monomers showed different physical and chemical properties.

The obtained catalytic monomers were used for the preparation of polymeric gel dots. For the construction of the MFR, the synthesized catalytic monomers were immobilized in the reactor as gel dots via photopolymerization using methyl methacrylate (MMA) or *N,N*-dimethylacrylamide (DMAM) as the gel-building component and *N,N*-methylenebis(acrylamide) (MBAM) or ethylene glycol dimethacrylate (EGDMA) as a crosslinker to obtain various compositions of gel dots, as given in Table 1.

**Figure 3:**
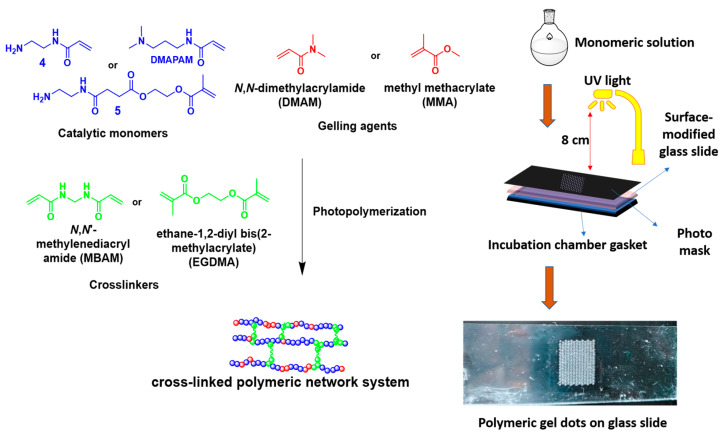
Preparation of polymeric gel dots via photolithography using various compositions.

Figure 3 shows the pictorial representation of the preparation of polymeric gel dots. These polymeric gel dots were prepared on microscopic glass slides, which were pre-modified with 3-(trichlorosilyl) propylmethacrylate. The modification of the glass slide facilitates reactive acrylate functionalities and those were used for the formation of a covalent connection between the polymeric networks and the glass slide during photopolymerization [21]. Furthermore, the parameters for photopolymerization, such as the concentration of the monomeric solution, distance between the UV light source and the sample plate, and the intensity of the UV light for different compositions are given in Table 1. After the preparation of the gel dots, the unreacted monomers were washed out and the gel dots were dried at room temperature for 2 d. Furthermore, neutralization of the gel dots was performed using triethylamine to remove HCL salts in the gels (Composition B and C). However, the gel dots prepared using composition A did not require any further purification because no salts were present in the system. Hence, these gel dots were directly used for the organocatalysis of the Knoevenagel reaction.

**Figure 4:**
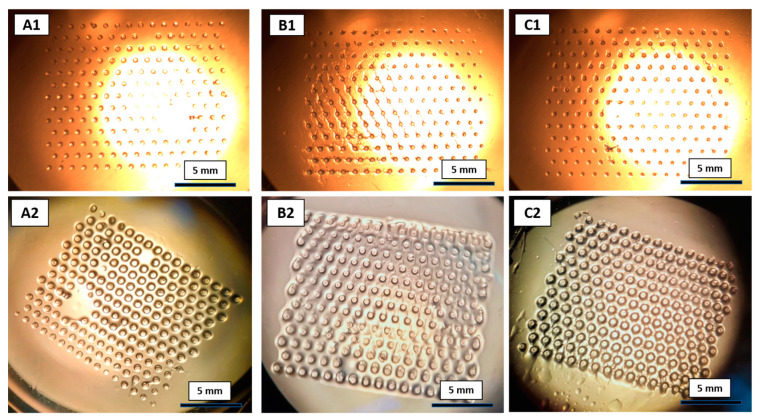
Optical microscopy images of gel dots with different compositions, A, B and C. Series 1 representing the dried gel dots and series 2 after swelling in the solvent mixture, DMSO: 2-propanol: water (*v*:*v*:*v* = 2:2:1), for 12 h.

In a previous work, the tertiary amine *N*-[3-(dimethylamino)propyl]acrylamide (DMAPAM) (of the composition A) was used as a catalyst for Knoevenagel reaction in the MFR, and was made with the same composition but with a surface area of 5.32 mm^2^ (the number of gel dots was 150) [9]. However, in our studies, the number of gel dots was increased up to 202 to increase the surface area of the total polymeric gel dots to 7.07 mm^2^. As a result, the catalytic activity of the MFR significantly increased. Similarly, the same number of polymeric gel dots were prepared using composition B and C. Moreover, in composition B and C, primary amines **4** and **5** were used as catalysts to perform the comparative studies of organocatalysis in the MFR. Optical microscopic images of various compositions, A, B and C, of gel dots before and after swelling for 12 h are shown in Figure 4. The diameter of the dry gel dots (A1, B1 and C1) were approximately 300 µm for all the compositions. These results were comparable to the diameter of the photomask of 350 µm. Correspondingly, the diameter of the swollen gels (A2, B2 and C2) increased up to 400 µm after the swelling in solvents, DMSO: 2-propanol: water (*v*:*v*:*v* = 2:2:1).

**Figure 5:**
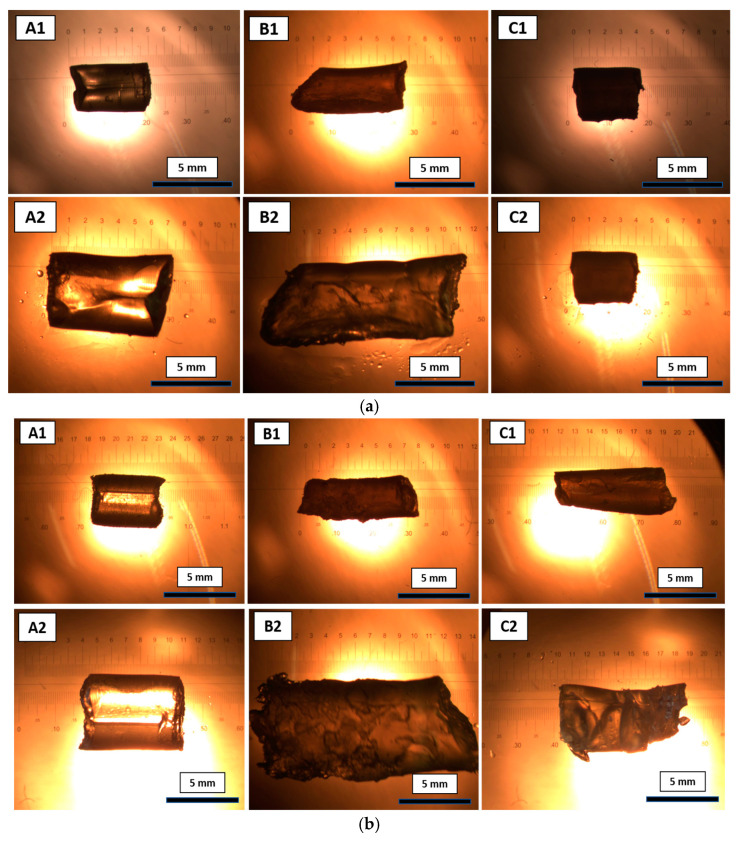
Optical microscopy images of polymeric gels with different compositions, A, B and C, of xerogels (1) and swollen gels (2) for 12 h. Swelling studies carried out with the solvent mixture, (**a**) DMSO: 2-propanol (1:1); (**b**) DMSO: 2-propanol: water (2:2:1).

The swelling of the gels provided a significant change in the organocatalysis [28], because, during the swelling process, the polymeric networks stretch to form a large mess-like structures. As a result, the accessibility of the catalytic sites increases for catalysis. Similarly, the catalytic activity of the gel dots increases with an increase in uptake of the reaction solvent. To evaluate the swelling properties of the gels, different compositions of monomer solutions were placed in Pasteur pipettes and polymerized with the parameters shown in Table 1. After polymerization, the polymeric networks were removed from the Pasteur pipette and purified using the protocol for gel dot preparation. After purification, the gels were dried at 50 °C until they obtained constant weights. Known weights and volumes of the dried gels (Figure 5A1–C1) were placed in sample vials and were swollen in the respective solvent mixtures, DMSO: 2-propanol (*v*:*v* = 1:1) and DMSO: 2-propanol: Water (*v*:*v*:*v* = 2:2:1), overnight. After 12 h of swelling, the swollen gels (Figure 5A2–C2) were taken out from the respective solvent mixture and the degree of swelling with respect to weight and volume was determined (Table 2). Approximately 200% swelling was observed for the gel composition A and B in solvent mixture DMSO: 2-propanol (*v*:*v* = 1:1) and DMSO: 2-propanol: water (*v*:*v*:*v* = 2:2:1), since the A and B gel compositions contained more hydrophobic components and flexible polymeric chains (Table 1). For gel composition C, significant swelling in the solvent mixture, DMSO: 2-propanol (*v*:*v* = 1:1), was not observed. Gel composition C contained more hydrophilic components (Table 1) and had a rigid short chain in the polymer network. Hence, the swelling properties of gel composition C could be increased with the addition of water to the solvent mixture (DMSO: 2-propanol: water (*v*:*v*:*v* = 2:2:1)). These results suggest that swelling is significantly influenced based on solvent mixture and the chemical composition of the gel. Likewise, the same compositions of gel dots showed the similar properties of swelling in the new solvent mixture (Figure 4). Consequently, the catalytic activity of the gel dots was influenced by the swelling properties.

The developed gel dots were introduced in the microfluidic reactor and the catalytic activity for the Knoevenagel reaction between various aldehydes and MN was studied. The general reactor setup was completed according to a previous report [9]. The square-shaped catalytically active polymeric gel dot arrays (total number 202) were placed in the reaction chamber with a height of 140 μm, forming a volume of 100 μL. Furthermore, to perform the continuously operated flow reactions, one end of the reactor was connected to syringes using PTFE capillary tubes (iD = 0.2 mm) as an inlet. Moreover, the two reactants were connected to a T-junction, which was used for the mixing of the two reactants in an equal molar ratio. The mixed solution was added into the reaction chamber through the inlet. The other side of the reactor was connected to the collector vial using a capillary tube as an outlet (Figure S1). The graphical representation of the MFR setup is shown in Figure 1. After the successful setup of the microfluidic reaction system, a Knoevenagel reaction between an aldehyde and MN was conducted using different amines as a catalyst. Initially, the polymeric gel dots were pre-swollen in the respective solvent mixture for 2 h with flow rate of 4 µL/min. This pre-swelling of the gel dots facilitates the expansion of the mess size in the polymeric networks. After the pre-swelling, the solution of the reactants in a 1:1 molar ratio with the respective solvent mixture were passed through the MFR with constant flow of 2 µL/min. The conversion of the reactants was determined via off-line NMR method with 8 h of collection of the eluent sample from the MFR after the residence time (45 min).

**Figure 6:**
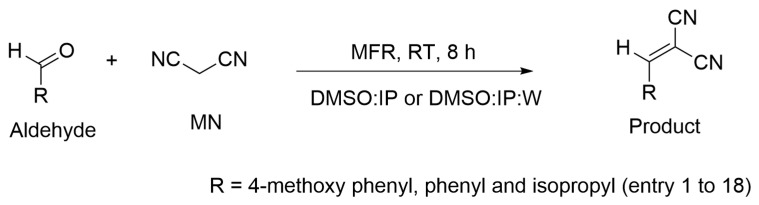
Scheme for the Knoevenagel reaction between different aldehydes and MN in the continues flow.

A Knoevenagel reaction was performed in the MFR with different aldehydes and MN (entry 1 to 18) using various polymeric gel dots as catalysts (Table 3). The scheme of the reaction is shown in Figure 6. The reaction was carried out in two different solvent mixtures, DMSO: 2-propanol *v*:*v* = 1:1) and DMSO: 2-propanol: water (*v*:*v*:*v* = 2:2:1). The conversion of the reaction was determined via off-line ^1^H NMR (Figures S2–S19) using the average collection of eluents after catalysis for 8 h at room temperature (Table 3). The conversion of the reaction was studied with a comparison of the various compositions of gel dots, A (tertiary amine) and B, C (primary amine). The poor conversion (2 to 4%) of the reaction between 4-nitro benzaldehyde and malononitrile without a catalyst in the MFR was taken as a reference. [9] The gel dots containing primary amines showed a better conversion of about 83–90% with aliphatic aldehyde (isobutyraldehyde) and 86–100% with aromatic aldehydes (4-methoxy benzaldehyde and benzaldehyde), when compared to the tertiary amines (52–59% with aliphatic aldehyde and 77–93% with aromatic aldehydes), because the nucleophilicity and basicity of the primary amines are higher than the tertiary amines in the polar solvents [29,30]. Furthermore, the catalytic efficacy of the Knoevenagel reaction was changed with solvent mixtures DMSO: 2-propanol (*v*:*v* = 1:1) and DMSO: 2-propanol: water (*v*:*v*:*v* =2:2:1). The conversion of the reaction was significantly higher with solvent mixture DMSO: 2-propanol: water (*v*:*v*:*v* = 2:2:1) compared to solvent mixture DMSO: 2-propanol *v*:*v* = 1:1), because protic solvents such as water accelerate the rate of reaction [31,32]. Moreover, the conversion drastically increased with gel composition C from about 20 to 70% in solvent mixture DMSO: 2-propanol: water (*v*:*v*:*v* = 2:2:1), compared to solvent mixture DMSO: 2-propanol *v*:*v* = 1:1), due to the increase in swelling properties of the gel dots as well as the acceleration of reaction rate with water.

## 3. Conclusions

The Knoevenagel reaction is one efficient reaction in organic chemistry for the synthesis of various organic molecules. In view of the improvement in organocatalysis, we have demonstrated the Knoevenagel reaction between various aldehydes and MN in a microfluidic system with different amines as catalysts. Primary-amine-based catalytic monomers **4** and **5** were synthesized and polymerized via photolithography to obtain polymeric gel dots with compositions A (tertiary amine (DMAPAM)), B and C (primary amine). Furthermore, the developed gel dots were inserted in to the microfluidic reactor and the conversion of the reaction between different aldehydes and MN in the respective solvent mixtures (DMSO: 2-propanol (*v*:*v* = 1:1) and DMSO: 2-propanol: water (*v*:*v*:*v* = 2:2:1)) was evaluated. The results suggested the conversion of the reaction increases with an increase in swelling of the gel dots, due to increased accessibility of the catalytic sites and the formation of capillary channels in the polymeric networks. Furthermore, the gel dots containing primary amines showed better conversion, about 83–90% with aliphatic aldehyde and 86–100% with aromatic aldehyde, when compared to the tertiary ones (52–59% with aliphatic aldehyde and 77–93% with aromatic aldehydes). This is due to the basicity of the primary amines being higher than the tertiary amines. Moreover, the higher conversion of the reaction was recorded via addition of water to the reaction mixture, because protic solvents facilitate a high conversion in the Knoevenagel reaction. Moreover, the basicity of the primary amine in the polar solvents was higher than in the tertiary amines. As a result, the catalytic activity of the gel dots with composition B and C showed better conversion compared to gel dot composition A. Hence, primary-amine-based gel dots as catalysts are more favorable than the tertiary-amine-based gel dots in the organocatalysis of the Knoevenagel reaction. The swelling of the gels and polarity of the reaction solvents showed a significant influence on organocatalysis and improved the efficiency of the MFR.

## 4. Materials and Methods

### 4.1. Materials

Ethylenediamine (99%) was purchased from Across Organics. Dimethylphenylphosphonite (98%), 3-(trichlorosilyl)propylmethacrylate 0%), *mono*-2-(methacryloyloxy)ethyl succinate, triethylamine (99%), methyl methacrylate (98%) (MMA), *N,N*-methylenebis(acrylamide) (MBAM), 1-hydroxybenzotriazol (HOBt) (99%), diisopropylethylamine (DIPEA) and 4-methoxy benzaldehyde were purchased from Sigma Aldrich. 2,4,6-Trimethylbenzoylchloride (>98%) and 2-butanone (99%) were purchased from Alfa Aesar. Dimethylsulfoxide (DMSO) (99.9%) was purchased from Grüssing GmbH. Benzaldehyde (98%), *N,N*-dimethylacrylamide (DMAM), 4N hydrogen chloride in 1,4-dioxane, malononitrile (MN), *N*-[3-(dimethylamino)propyl]acrylamide (DMAPAM) (98%) and 1-ethyl-3-(3-dimethylaminopropyl)carbodiimide hydrochloride (EDC·HCl) were purchased from Tokyo Chemical Industries (TCI). Ethylene glycol diacrylate was obtained from Merck. Di-*tert*-butyl dicarbonate (>97%) and isobutyraldehyde (98%) was obtained from Thermo Scientific. Aqueous ammonia (25%), hydrogen peroxide (30%), 2-propanol (technical grade), methanol (technical grade), ethanol (technical grade), dichloromethane (DCM) (HPLC), citric acid, sodium chloride, aluminium oxide and ethyl acetate were obtained from Stockmeier Chemie, Bielefeld. Sodium hydrogen carbonate, magnesium sulfate and sodium sulfate were procured from VWR International GmbH Chemicals, Langenfeld. Argon 5.0, Wohning-Gas GmbH, Paderborn.Lithium phenyl-2,4,6-trimethylbenzoyl-phosphinate (LPTMBP) as an initiator was synthesized as per reported procedure [33].

### 4.2. Analysis

^1^H and ^13^C NMR (Nuclear Magnetic Resonance) spectra were recorded on a Bruker “Ascent 700” spectrometer using deuterated solvents. NMR signals were recorded with respect to the reference signal, tetramethyl silane (TMS). The electrospray ionization mass spectrometry (ESI-MS) measurements were carried out on a “Synapt-G2 HDMS” mass spectrometer from Waters in combination with a time of flight (TOF) analyzer. The images of the gels were taken using a “Hund Wetzlar” microscope and FLQ 150 M cold light source. The images were captured with an iDS uEye camera and uEye Cockpit software. The diameters of the gels were measured using a microscopic ruler, PYSER-SGI Ltd.

### 4.3. Synthesis of Catalytic Monomers

#### 4.3.1. Synthesis of tert-butyl N-(2-aminoethyl)carbamate (1)

A solution of ethylenediamine (600 mmol) in 600 mL of DCM was placed in a 1000 mL round-bottom flask and a solution of di-*tert*-butyldicarbonate (100 mmol) in 200 mL of DCM was added dropwise to the above solution for 3 h at room temperature using a dropping funnel. After addition, the reaction mixture was further stirred for 24 h at the same temperature. The reaction was monitored via thin layer chromatography (TLC) using DCM/MeOH, 9:1, After the completion of the reaction, the reaction mixture was concentrated under reduced pressure. The crude oily product was dissolved in aqueous 2M sodium carbonate (300 mL) and extracted thrice with DCM (200 mL). The organic layer was washed twice with 2M sodium carbonate (200 mL) and dried over anhydrous MgSO_4_. The solvent was evaporated under reduced pressure to yield pure product **1** (light yellow oil)

Yield: 87%. ^1^H NMR (700 MHz, CDCl_3_): δ (ppm) = 1.38 (br s, 9H, CH_3_), 1.73 (br s, 2H, NH_2_), 2.75 (t, ^3^*J*_HH_ = 5.93 Hz, 2H, CH_2_), 3.12 (t, ^3^*J*_HH_ = 5.30 Hz, 2H, CH_2_), 5.14 (br s, 1H, NH). ^13^C NMR (176 MHz, CDCl3): δ (ppm) = 28.2, 41.6, 43.1, 79.0 and 156.2. ESI-MS (m/z): C_7_H_17_N_2_O_2_^+^ [M + H]^+^, mass calculated: 161.1200 Da, mass found: 161.1312 Da.

#### 4.3.2. Synthesis of tert-butyl N-[2-(prop-2-enoylamino)ethyl]carbamate (2)

Compound **1** (6.3 mmol) was placed in a 250 mL two-necked round-bottom flask and dissolved in DCM (120 mL). Triethylamine (9.3 mmol) was added to the reaction mixture and then stirred for 30 min at room temperature. Later, the reaction mixture was cooled to 0 ^⁰^C and then acryloyl chloride (9.4 mmol) was added to the reaction mixture in a dropwise manner for 20 min. After addition, the reaction mixture was stirred at room temperature overnight. After the completion of the reaction, the reaction mixture was filtered and the precipitate was washed with DCM (50 mL). The filtrate was washed twice with brine solution (200 mL), saturated sodium carbonate (100 mL) and DI water (200 mL). The organic layer was dried with anhydrous sodium sulfate, and then concentrated using a rotatory evaporator. The crude product was recrystallized with toluene (200 mL) overnight. Next, the crystals were separated by decanting the toluene solution and the pure crystals were washed twice with pure toluene (50 mL) and then dried under high vacuum for 6 h to yield colorless solid **2**.

Yield: 57%. ^1^H NMR (700 MHz, *DMSO-d*_6_): δ (ppm) = 1.37 (s, 9H, CH_3_), 3.00 (t, ^3^J_HH_ = 6.07 Hz, 2H, CH_2_), 3.14 (t, ^3^J_HH_ = 5.90 Hz, 2H, CH_2_), 5.57 (dd, ^3^J_HH_ = 10.17, ^2^J_HH_ = 2.17 Hz, 1H, CH_2_), 6.06 (dd, ^3^J_HH_ = 17.09, ^3^J_HH_ = 10.20 Hz, 1H, CH), 6.18 (dd, ^3^J_HH_ = 17.09, ^2^J_HH_ = 2.17 Hz, 1H, CH), 6.82 (br s, 2H,), 8.10 (br s, 1H, NH). ^13^C NMR (176 MHz, *DMSO-d*_6_): δ (ppm) = 28.2, 38.7, 40.0, 77.6, 124.9, 131.8, 155.6, 164.7. ESI-MS (m/z): C_10_H_18_N_2_O_3_^+^ [M + Na]^+^, mass calculated: 237.1215 Da, mass found: 237.1223 Da.

#### 4.3.3. Synthesis of 2-(methacryloyloxy)ethyl-4-((2-((tert-butoxycarbonyl)amino) ethyl)amino)-4-oxobutanoate (3)

A mixture of EDC.HCl (86 mmol), HOBt (86 mmol) and *mono*-2-(methacryloyloxy)ethyl succinate (86 mmol) were placed in a 500 mL round-bottom flask and dissolved in DCM (250 mL). Then, the reaction mixture was stirred for 20 min under an argon atmosphere. Compound **1** (78 mmol) and DIPEA (86 mmol) were added to the reaction mixture and the reaction mixture was stirred at room temperature for 24 h. After the completion of the reaction, the reaction mixture was diluted with DCM (100 mL) and the reaction mixture was washed twice with water (150 mL), saturated NaHCO_3_ (150 mL)_,_ saturated citric acid (150 mL) and brine solution (150 mL). Furthermore, the organic phase was passed through a hydrophobic filter and the solvent was removed by a rotary evaporator. The crude product was further purified with silica column chromatography using a 1:9 volume ratio of ethyl acetate and *n*-hexane to a 9:1 volume ratio of ethyl acetate and *n*-hexane as eluent. The organic solvent was evaporated under reduced pressure to obtain pure colorless solid **3** as a product.

Yield: 71%. ^1^H-NMR (700 MHz, CDCl_3_): δ (ppm) = 1.42 (s, 9H, CH_3_), 1.93 (s, 3H, CH_3_), 2.48 (t, ^3^J_HH_ = 6.9 Hz, 2H, CH_2_), 2.68 (t, ^3^J_HH_ = 6.9 Hz, 2H, CH_2_), 3.24 (t, ^3^J_HH_ = 6.1 Hz, 2H, CH_2_), 3.34 (t, ^3^J_HH_ = 6.1 Hz, 2H, CH_2_), 4.3 (m, 4H, CH_2_), 4.98 (s, 1H, NH), 5.58 (t, ^3^J_HH_ = 1.5 Hz, 1H, CH_2_), 6.11 (s, 1H, CH_2_), 6.52 (s, 1H, NH). ^13^C-NMR (126 MHz, CDCl_3_): δ (ppm) = 18.5, 28.5, 29.6, 30.1, 40.5, 40.8, 62.5, 62.6, 79.9, 126.4, 136.1, 157.0, 167.4, 172.3, 172.9. ESI-MS (m/z): C_17_H_28_N_2_O_7_^+^ [M + Na]^+^, mass calculated: 395.1794 Da, mass found: 395.1813 Da.

#### 4.3.4. Synthesis of N-(2-aminoethyl)prop-2-enamide. hydrochloride (4)

Compound **2** (2.3 mmol) was placed in a 50 mL of round-bottom flask and 4M HCl in 1,4-dioxane solution (20 mL) was added and then stirred vigorously at room temperature overnight. After the completion of the reaction, the pure product was concentrated under reduced pressure. The crude product was washed with diethyl ether and dried at high vacuum for 6 h to obtain a yellowish solid **4**.

Yield: 90%.^1^H NMR (700 MHz, *DMSO-d*_6_): δ (ppm) = 2.88 (t, ^3^J_HH_ = 5.9 Hz, 2H, CH_2_), 3.38 (t, ^3^J_HH_ = 6.01 Hz, 2H, CH_2_), 5.62 (dd, ^3^J_HH_ = 10.23, ^2^J_HH_ = 1.75 Hz, 1H, CH_2_), 6.12 (dd, ^3^J_HH_ = 17.12, ^2^J_HH_ = 10.23 Hz,1H, CH), 6.22 (dd, ^3^J_HH_ = 17.12, ^2^J_HH_ = 1.80 Hz,, 1H, CH), 8.16 (br s, 3H, H_3_N^+^), 8.56 (br s, 1H, NH). ^13^C NMR (176 MHz, *DMSO-d*_6_): δ (ppm) = 36.5, 38.4, 66.35, 125.5, 131.5, 165.2. ESI-MS (m/z): C_5_H_11_N_2_O^+^ [M–Cl]^+^, mass calculated: 115.0871 Da, mass found: 115.0864 Da.

#### 4.3.5. Synthesis of 2-(methacryloyloxy)ethyl-4-((2-amino) ethyl)amino)-4-oxobutanoate. hydrochloride (5)

Compound 3 (55 mmol) was placed in a 250 mL round-bottom flask and 4M HCl 1,4-dioxane solution (116 mL) was added. Then, the reaction mixture was stirred at room temperature overnight. The resulting suspension was filtered and the filtrate was concentrated by a rotary evaporator. Furthermore, the product was dried under a high vacuum to obtain a pale yellow oil **5**

Yield: 91% ^1^H-NMR (700 MHz, *DMSO-d*_6_): δ (ppm) = 1.87 (s, 3H, CH_3_), 2.39 (t, ^3^J_HH_ = 7.0 Hz, 2H, CH_2_), 2.52 (t, ^3^J_HH_ = 7.1 Hz, 2H, CH_2_), 2.82 (m, ^3^J_HH_ = 6.0 Hz, 2H, CH_2_), 3.29 (m, ^3^J_HH_ = 6.0 Hz, 2H, CH_2_), 4.26 (m, 4H, CH_2_), 5.69 (s, 1H, CH_2_), 6.02 (s, 1H, CH_2_), 8.17 (s, 3H, H_3_N^+^), 8.27 (s, NH). ^13^C-NMR (176 MHz, *DMSO-d*_6_): δ (ppm) = 18.0, 28.8, 29.8, 36.4, 38.5, 61.8, 62.5, 126.2, 135.6, 166.4, 171.3, 172.3. ESI-MS (m/z): C_12_H_21_N_2_O_5_^+^ [M–Cl]^+^, mass calculated: 273.1450 Da, M—ass found: 273.1439 Da.

### 4.4. Modification of Glass Slides

The modification of glass slides (7.6 cm × 2.6 cm) was performed as per the reported procedure [9]. Briefly, the microscopic glass slides (10 numbers) were washed with 2-propanol, water and ethanol under sonication for 10 min each. The washed glass slides were oxidized using a mixture of deionized water (100 mL), 25% aqueous ammonia solution (20 mL) and 35% H_2_O_2_ (20 mL) at 70°C for 10 min in the presence of sonication. The oxidized glass slides were dried using nitrogen gas and stored in the desiccator. Further modification of the glass substrate, 3-(trichlorosilyl)propylmethacrylate (150 µL), was added to the desiccator and a vacuum (50 mbar) was applied for 2 h to form a thin layer of silyl acrylate on the glass slide. These modified glass slides were used for the preparation of gel dots.

### 4.5. Preparation of Gel Dots Using Various Compositions of Gel Dots

The polymeric networks of various compositions (Table 1) were prepared using photopolymerization using an “Omnicure^®^ S1500” UV-Lamp from Lumen Dynamics, as shown in Figure 3. A monomer solution (15 mmol) containing a catalyst, gel-forming agent (*N,N*-dimethylacrylamide or methyl methacrylate) and crosslinker (*N,N*-methelene(bisacrylamide) or ethylenediacrylate) was prepared in DI water (1.7 mL). To the monomer solution, initiator (LPTMBP) (17.5 mg/mL) was added and stirred for 2 h in the dark. Furthermore, the monomer solution (200 µL) was placed in an incubation chamber gasket, and then the monomer solution was covered with a modified microscopic glass slide. Next, the photopolymerization mask (202 dots) was placed on the top of glass slide as a sandwich. The sandwich system was irradiated with various parameters as given in Table 1. After polymerization, the gasket chamber was placed in DI water (200 mL) to wash the polymer gel dots. Furthermore, the gel dots were washed with 2-propanol overnight and then dried at room temperature for 2 days. The neutralization of the gel dots was performed to remove the salts (HCl) in the gel composition. The gel dots were washed for 1 h each with a mixture of methanol/triethylamine (9:1) ratio (100 mL) and tetrahydrofuran/ triethylamine (9:1) ratio (100 mL), followed by pure solvents (tetrahydrofuran and methanol) (100 mL). The purified gel dots were dried at room temperature.

### 4.6. Swelling Studies of Gels

The swelling properties of gel compositions A, B, and C were performed by solvent uptake studies and changes in volume before and after swelling in the reaction solvent mixtures, DMSO/2-propanol (*v*:*v* = 1:1) and DMSO/2-propanol/water (*v*:*v*:*v* = 2:2:1). The monomeric solution was placed in a Pasteur pipette and irradiated under UV light with the parameters as shown in Table 1. After polymerization, the gels were stored at room temperature overnight and the gels were taken out from the Pasteur pipette. The cylindrical gels were washed with water and 2-propanol overnight and dried at 40 °C until stable weight was obtained. The dried gels were swollen in the respective solvent mixture overnight. However, the gels with composition B and C were used for swelling studies after neutralization, as mentioned in the above protocol for polymer gel dots. The weights of the gels (before and after swelling) were measured to determine the solvent uptake. The percentage of solvent uptake was calculated using Equation (1) for all the gel compositions.

(1) $$Percentage\;of\;solvent\;uptake=\frac{Wt.Sg-Wt.Dg}{Wt.Dg}\;100$$

where,

Wt.Sg = Weight of the swollen gel (mg)

Wt.Dg = Weight of the dry gel (mg)

Furthermore, the volume of the cylindrical gels before and after swelling was calculated using Equation (3). The length and width of the gels were recorded using a microscopic ruler, which were shown in the optical images of the gels (Figure 5). The swelling ratio was calculated using Equation (2).

(2) $$Degree\;of\;swelling\;\left(Ds\right)=\frac{Vs}{Vd}\;$$

where,

Vs = Volume of the swollen gel (mm^3^)

Vd = Volume of the dry gel (mm^3^)

The volume of the cylindrical gel was calculated by following Equation (3).

V = πr^2^ × h (3)

where,

V = Volume of the gel (mm^3^)

r = Radius of cylindrical gel (mm)

h = Height of the gel (mm)

### 4.7. Microfluidic Reactor Assembly

The assembly of the microfluidic reactor was conducted as shown in the schematic diagram, Figure 1. The flow rate of the reactant solution was controlled using a “Legato 200” syringe pump (KDScientific) which was equipped with two syringes and a “Hamilton 1000 series” as a reservoir. The design of the reactor was as described by Simon et al. and the reactor was covered with self-made alumina holders used to connect the imprinted PTFE layer with the microfluidic flow system [34]. The microscopic glass slide with polymeric gel dots was inserted into the MFR. The polymeric gel dots were covered with an imprinted PTFE layer, with the height of the channels being approximately 140 μm. This sandwich system was fixed by screws with torque of 8 cN/m. Furthermore, the reactor, syringe pump and collector vial were connected to the PTFE capillary tubes (iD = 0.2 mm) from Fisher Scientific.

### 4.8. Microfluidic Flow Reaction

Initially, the polymeric gel dots were pre-swollen with the respective solvent mixture (DMSO/2-propanol (*v*:*v* = 1:1) and DMSO/2-propanol/water (*v*:*v*:*v* = 2:2:1)) without reactants for 2 h with the flow rate of 2.0 µL/min per syringe (This meant the total flow rate in the reactor was 4.0 µL/min). Overall, the syringe pump was loaded with the respective reactant (aldehyde and MN) solution in the respective solvent mixture and the flow rate was adjusted to 2.0 µL/min. The product was collected for 8 h after the residence time (45 min) and the conversion of the reaction was determined. All the microfluidic flow reactions were performed at room temperature.

### 4.9. Determination of Conversion

The conversion of the reaction was determined via off-line ^1^H NMR spectroscopy. Briefly, 100 µL of the collected outlet flow solution was placed in a sample vial and diluted with 500 µL of DMSO-d_6_. Thereafter, the samples were scanned using an NMR spectrometer. The conversion of the reaction was estimated using the integral of the product peak (b) with the respective aldehyde (a) peak integrals (Figure S2–S19).

### 4.10. Knoevenagel Reaction between Different Aldehydes with Malononitrile in the Continuous Flow

#### 4.10.1. Synthesis of 2-(4-methoxybenzylidene) malononitrile (entry **1–6**)

A solution of MN (16 mmol) and 4-nitro benzaldehyde (16 mmol) was prepared separately in 8 mL of the respective solvent mixtures, (DMSO/2-propanol (*v*:*v* = 1:1) or DMSO/2-propanol/water (*v*:*v*:*v* = 2:2:1), as mentioned in Table 3. The prepared solutions were placed into a 10 mL of syringe and fixed to the syringe pump, as shown in Figure S1, at a flow rate of 1 µL/min per syringe. The overall flow rate of the MFR with the two reactant solutions was 2 µL/min. The product was collected from the MFR in a vial as eluent at room temperature for 8 h (excluding residence time for 45 min). The collected product was diluted three times with water and the product was extracted with diethyl ether. The organic layer was concentrated using a rotatory evaporator to obtain a yellow -colored solid, 2-(4-methoxybenzylidene) malononitrile.

^1^H NMR (700 MHz, *DMSO-d*_6_): δ (ppm) = 3.89 (s, 3H, CH_3_), 7.18 (d, ^3^J_HH_ = 8.9 Hz, 2H, CH), 7.97 (d, ^3^J_HH_ = 8.9 Hz, 2H, CH) and 8.38 (s, 1H). ^13^C NMR (176 MHz, *DMSO-d*_6_): δ (ppm) = 56.0, 113.9, 115.3, 124.4, 133.6 and 160.5. ESI-MS (m/z): C_11_H_8_N_2_O^+^ [M]^+^, mass calculated: 184.0613 Da, mass found: 184.0623 Da.

#### 4.10.2. Synthesis of 2-benzylidenemalononitrile (entry **7–12**)

A solution of MN (16 mmol) and benzaldehyde (16 mmol) was prepared separately in 8 mL of respective solvent mixtures, (DMSO/2-propanol (*v*:*v* = 1:1) or DMSO/2-propanol/water (*v*:*v*:*v* = 2:2:1) (Table 3). The reactant solutions were placed in a 10 mL syringe and injected into the syringe pump (Figure S1) at a flow rate of 1 µL/min per syringe. The overall flow rate of MFR with two reactant solutions was 2 µL/min. The product was collected in a vial from the MFR at room temperature for 8 h (excluding a residence time of 45 min) and diluted with water (three times). The product was extracted with diethyl ether and the organic layer was concentrated under reduced pressure to yield a colorless solid, 2-benzylidenemalononitrile.

^1^H NMR (700 MHz, *DMSO-d*_6_): δ (ppm) = 7.62 (t, ^3^J_HH_ = 7.8 Hz, 2H, CH), 7.7 (t, ^3^J_HH_ = 7.5 Hz, 1H, CH), 7.95 (d, ^3^J_HH_ = 7.6 Hz, 2H, CH) and 8.54 (s, 1H). ^13^C NMR (176 MHz, *DMSO-d*_6_): δ (ppm) = 81.6, 113.2, 114.2, 129.5, 130.5, 131.3, 134.4 and 161.6. ESI-MS (m/z): C_10_H_6_N_2_^+^ [M ]^+^, mass calculated: 154.0531 Da, mass found: 154.0527 Da.

#### 4.10.3. Synthesis of 2-(2-methylpropylidene) malononitrile (entry **13–18**)

A solution of MN (16 mmol) and isobutyraldehyde (16 mmol) was prepared separately in 8 mL of the respective solvent mixtures, (DMSO/2-propanol (*v*:*v* = 1:1) or DMSO/2-propanol/water (*v*:*v*:*v* = 2:2:1), as given in Table 3. The reactant solutions were placed in a 10 mL syringe and injected into the syringe pump (Figure S1) at a flow rate of 1 µL/min per syringe. Subsequently, the overall flow rate of MFR with the two reactants was 2 µL/min. The product was collected in a vial from the MFR for 8 h at room temperature (excluding a residence time of 45 min) and diluted with water (three times). Then, the product was extracted with diethyl ether. The organic layer was concentrated under a reduced pressure to obtain a brown-colored oil, 2-(2-methylpropylidene) malononitrile.

^1^H NMR (700 MHz, *DMSO-d*_6_): δ (ppm) = 1.10 (d, ^3^J_HH_ = 6.6 Hz, 6H, CH_3_), 2.81 (m, 1H, CH) and 7.81 (d, ^3^J_HH_ = 10.3 Hz, 1H, CH). ^13^C NMR (176 MHz, *DMSO-d*_6_): δ (ppm) = 20.4, 32.7, 81.3, 111.2, 112.8 and 177.3. ESI-MS (m/z): C_7_H_8_N_2_^+^ [M]^+^, mass calculated: 120.0687 Da, mass found: 120.0680 Da.

## Figures and Tables

**Table 1:** Preparation of the various polymer gel dot compositions.

Composition Code	Catalytic Monomer	Complimentary Monomer	Crosslinker	UV Irradiation Time (s)	UV Intensity (W)	Number of Gel Dots
A	DMAPAM	DMAM	MBMA	8	0.42	202
B	**5**	MMA	EGDMA	14	0.42	202
C	**4**	DMAM	MBMA	90	1.28	202

**Table 2:** Degree of swelling with respect to weight and volume for various gel compositions for 12 h.

Composition Code	Solvent Uptake (%)		Volume Degree of Swelling (mm^2^)	
	* DMSO:IP	* DMSO:IP:W	* DMSO:IP	* DMSO:IP:W
A	266 ± 0.1	211 ± 14	2.25	1.85
B	220 ± 21	165 ± 18	2.10	2.08
C	23 ± 5	177 ± 19	1.00	1.61

* Here, DMSO:IP (dimethyl sulfoxide: 2-propanol (*v*:*v* = 1:1)) and DMSO:IP:W (dimethyl sulfoxide: 2-propanol: water (*v*:*v*:*v* = 2:2:1)).

**Table 3:** Conversion of the reaction with different aldehydes and MN in continuous flow.

Entry	Reactant 1	Reactant 2	Solvent Mixture	Conversion (%) with Different Compositions		
				Gel Dot Composition A	Gel Dot Composition B	Gel Dot Composition C
1–3	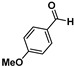 MBA	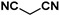 MN	* DMSO:IP	77	87	67
4–6	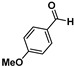 MBA	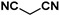 MN	* DMSO:IP:W	93	100	98
7–9	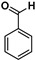 BA	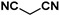 MN	* DMSO:IP	38	99	82
10–12	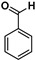 BA	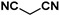 MN	* DMSO:IP:W	81	86	98
13–15	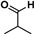 IBA	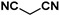 MN	* DMSO:IP	59	75	54
16–18	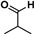 IBA	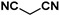 MN	* DMSO:IP:W	52	83	91

* (dimethyl sulfoxide: 2-propanol (*v*:*v* = 1:1)) and DMSO:IP:W (dimethyl sulfoxide: 2-propanol: water (*v*:*v*:*v* = 2:2:1)). Entry 1–3 are the reactions between 4-methoxybenzaldehyde (MBA) and MN using gel dot composition A, B and C as catalyst in solvent mixture DMSO:IP. Entry 4–6 are the reactions between MBA and MN using gel dot composition A, B and C as catalyst in solvent mixture DMSO:IP:W. Entry 7–9 are the reactions between benzaldehyde (BA) and MN using gel dot composition A, B and C as catalyst in solvent, DMSO:IP. Entry 10–12 are the reactions between BA and MN using gel dot composition A, B and C as catalyst in solvent mixture DMSO:IP:W. Entry 13–15 are the reactions between isobutyraldehyde (IBA) and MN using gel dot composition A, B and C as catalyst in solvent mixture DMSO:IP. Entry 16–18 are the reactions between IBA and MN using gel dot composition A, B and C as catalyst in solvent mixture DMSO:IP:W.

## Data Availability

Not applicable.

## Supplementary Materials

The following supporting information can be downloaded at: https://www.mdpi.com/article/10.3390/gels9030171/s1, Figure S1: Microfluidic reaction setup; Figure S2: ^1^H spectra for determination of conversion of the reaction between 4-methoxy benzaldehyde and malononitrile using polymeric gel composition A in solvent mixture, DMSO:IP (*v*:*v*=1:1) for 8 h; Figure S3: ^1^H spectra for determination of conversion of the reaction between 4-methoxy benzaldehyde and malononitrile using polymeric gel composition B in solvent mixture, DMSO:IP (*v*:*v*=1:1) for 8 h; Figure S4: ^1^H spectra for determination of conversion of the reaction between 4-methoxy benzaldehyde and malononitrile using polymeric gel composition C in solvent mixture, DMSO:IP (*v*:*v*=1:1) for 8 h; Figure S5: ^1^H spectra for determination of conversion of the reaction between 4-methoxy benzaldehyde and malononitrile using polymeric gel composition A in solvent mixture, DMSO:IP:W (*v*:*v*:*v*=2:2:1) for 8 h; Figure S6: ^1^H spectra for determination of conversion of the reaction between 4-methoxy benzaldehyde and malononitrile using polymeric gel composition B in solvent mixture, DMSO:IP:W (*v*:*v*:*v*=2:2:1) for 8 h; Figure S7: ^1^H spectra for determination of conversion of the reaction between 4-methoxy benzaldehyde and malononitrile using polymeric gel composition C in solvent mixture, DMSO:IP:W (*v*:*v*:*v*=2:2:1) for 8 h; Figure S8: ^1^H spectra for determination of conversion of the reaction between benzaldehyde and malononitrile using polymeric gel composition A in solvent mixture, DMSO:IP (*v*:*v*=1:1) for 8 h; Figure S9: ^1^H spectra for determination of conversion of the reaction between benzaldehyde and malononitrile using polymeric gel composition B in solvent mixture, DMSO:IP (*v*:*v*=1:1) for 8 h; Figure S10: ^1^H spectra for determination of conversion of the reaction between benzaldehyde and malononitrile using polymeric gel composition C in solvent mixture, DMSO:IP (*v*:*v*=1:1) for 8 h; Figure S11: ^1^H spectra for determination of conversion of the reaction between benzaldehyde and malononitrile using polymeric gel composition A in solvent mixture, DMSO:IP.W (*v*:*v*:*v*=2:2:1) for 8 h; Figure S12: ^1^H spectra for determination of Conversion of reaction between benzaldehyde and malononitrile using polymeric gel composition B in solvent mixture, DMSO:IP.W (*v*:*v*:*v*=2:2:1) for 8 h; Figure S13: ^1^H spectra for determination of Conversion of reaction between benzaldehyde and malononitrile using polymeric gel composition C in solvent mixture, DMSO:IP.W (*v*:*v*:*v*=2:2:1) for 8 h; Figure S14: ^1^H spectra for determination of conversion of reaction between iso-butyraldehyde and malononitrile using polymeric gel composition A in solvent mixture, DMSO:IP (*v*:*v*=1:1) for 8 h; Figure S15: ^1^H spectra for determination of conversion of reaction between iso-butyraldehyde and malononitrile using polymeric gel composition B in solvent mixture, DMSO:IP (*v*:*v*=1:1) for 8 h; Figure S16: ^1^H spectra for determination of conversion of reaction between iso-butyraldehyde and malononitrile using polymeric gel composition C in solvent mixture, DMSO:IP (*v*:*v*=1:1) for 8 h; Figure S17: ^1^H spectra for determination of conversion of reaction between iso-butyraldehyde and malononitrile using polymeric gel composition A in solvent mixture, DMSO:IP.W (*v*:*v*:*v*=2:2:1) for 8 h; Figure S18: ^1^H spectra for determination of conversion of reaction between iso-butyraldehyde and malononitrile using polymeric gel composition B in solvent mixture, DMSO:IP.W (*v*:*v*:*v*=2:2:1) for 8 h; Figure S19: ^1^H spectra for determination of conversion of reaction between iso-butyraldehyde and malononitrile using polymeric gel composition C in solvent mixture, DMSO:IP.W (*v*:*v*:*v*=2:2:1) for 8 h; Figure S20: Conversion of the reaction with malononitrile and different aldehydes (i) 4-methoxybenzaldehyde, (ii) benzaldehyde and (iii) isobutyraldehyde using various polymeric gel dots compositions A, B and C for 8 h.
