# Supplementary material for: Polymeric Networks Containing Amine Derivatives as Organocatalysts for Knoevenagel Reaction within Continuously Driven Microfluidic Reactors
Source: Gels. 2023 Feb 21;9(3):171. doi: 10.3390/gels9030171 (PMC10048661; doi:10.3390/gels9030171)
Supplement: Supplementary file 1 [file gels-09-00171-s001.zip › gels-2229603-supplementary.pdf]

gels

Article

Polymeric Networks Containing Amine Derivatives as Organocatalysts for Knoevenagel Reaction within Continuously Driven Microfluidic Reactors

Naresh Killi, Julian Bartenbach and Dirk Kuckling *

Department of Chemistry, Faculty of Science, Paderborn University, Warburger Str. 100, 33098 Paderborn, Germany
* Correspondence: dirk.kuckling@uni-paderborn.de

gels

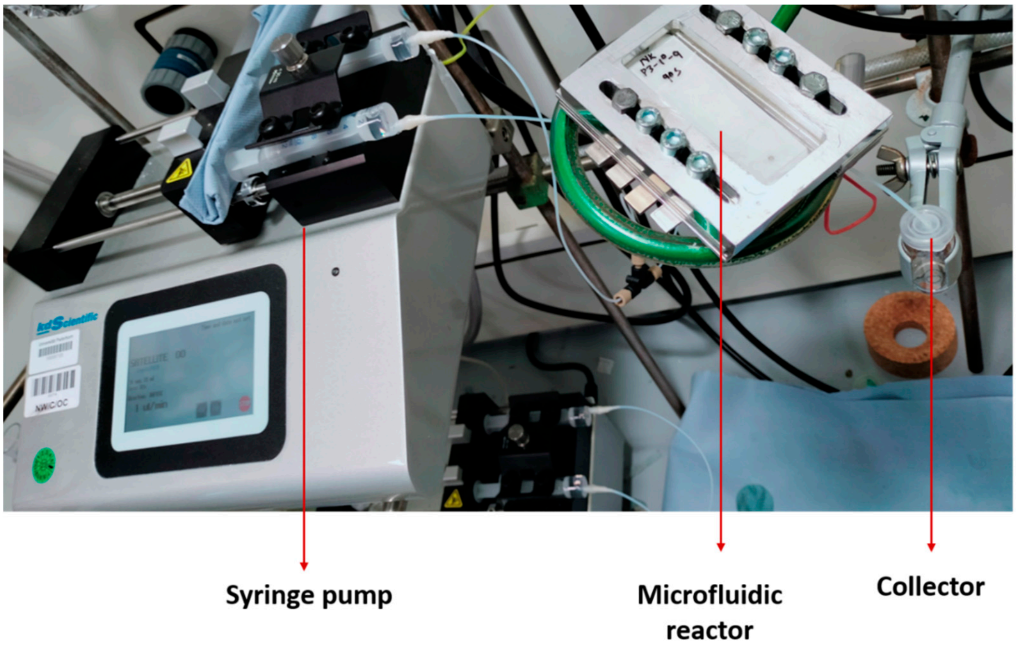

Figure S1: Microfluidic reaction setup.

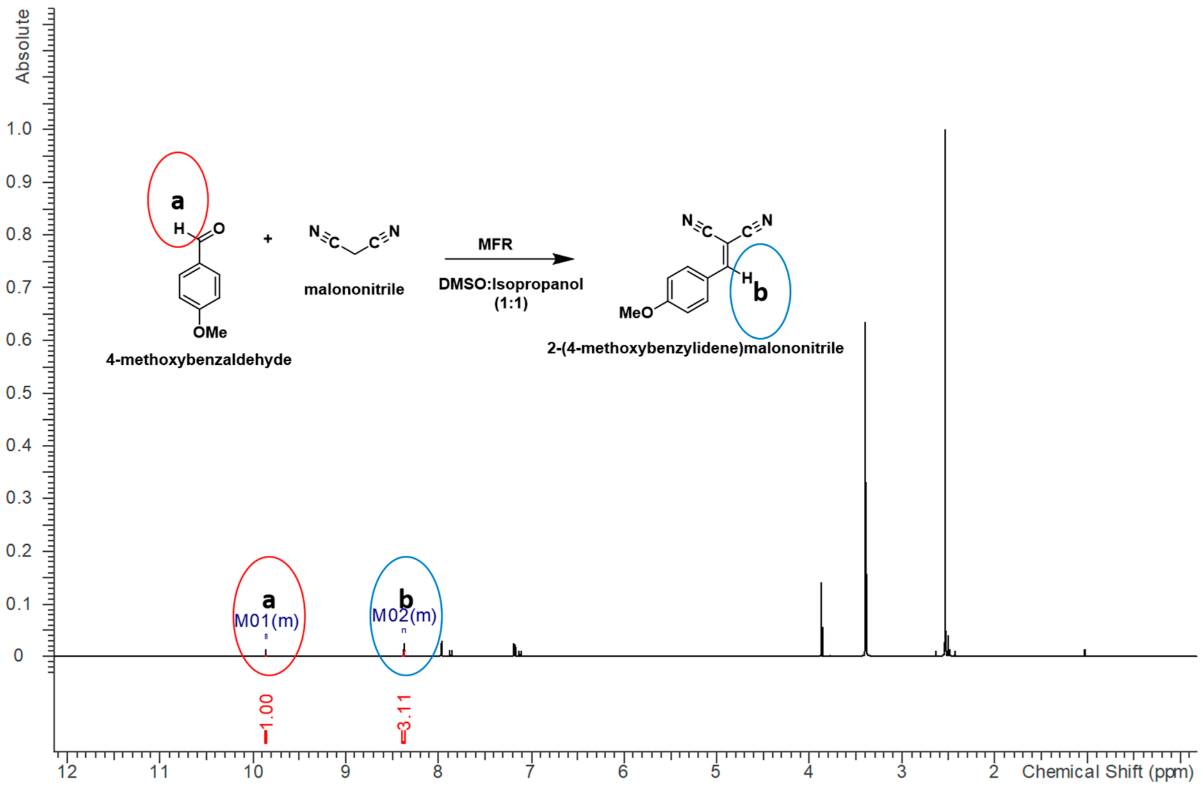

Figure S2: ^{1}H spectra for determination of conversion of the reaction between 4-methoxy benzaldehyde and malononitrile using polymeric gel composition A in solvent mixture, DMSO:IP (v:v=1:1) for 8 h.

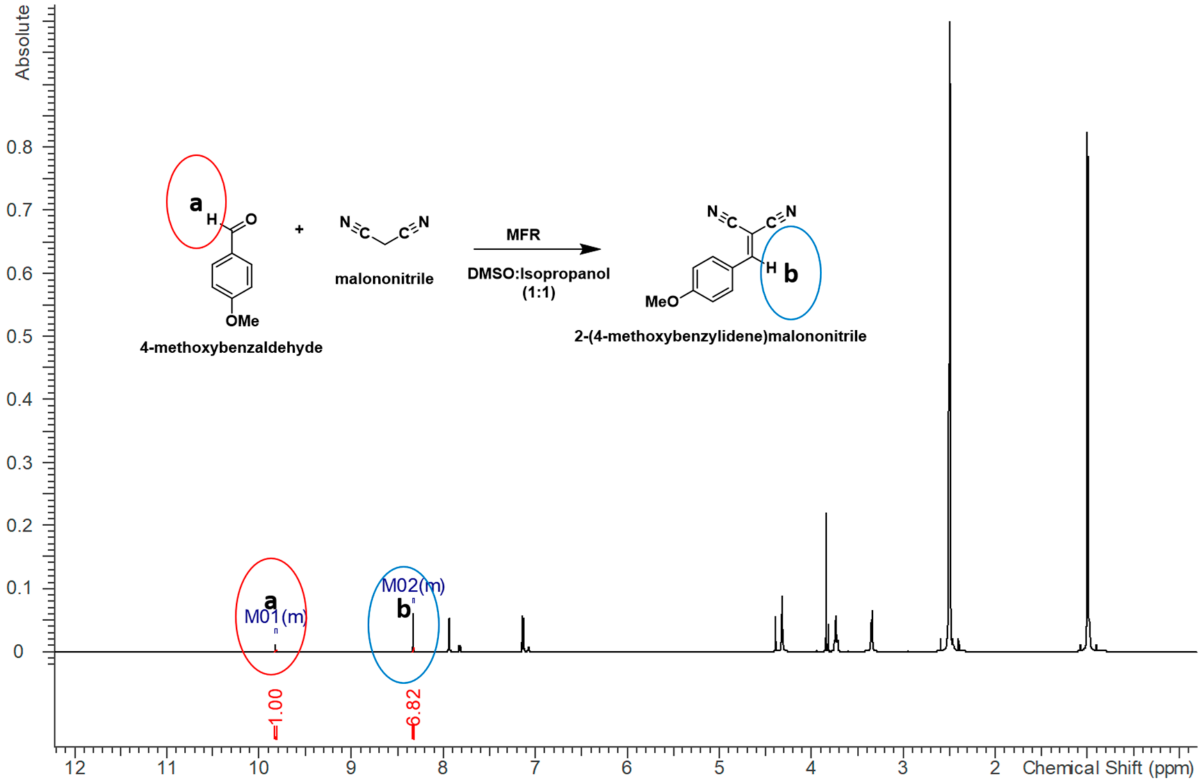

Figure S3: ^{1}H spectra for determination of conversion of the reaction between 4-methoxy benzaldehyde and malononitrile using polymeric gel composition B in solvent mixture, DMSO:IP (v:v=1:1) for 8 h.

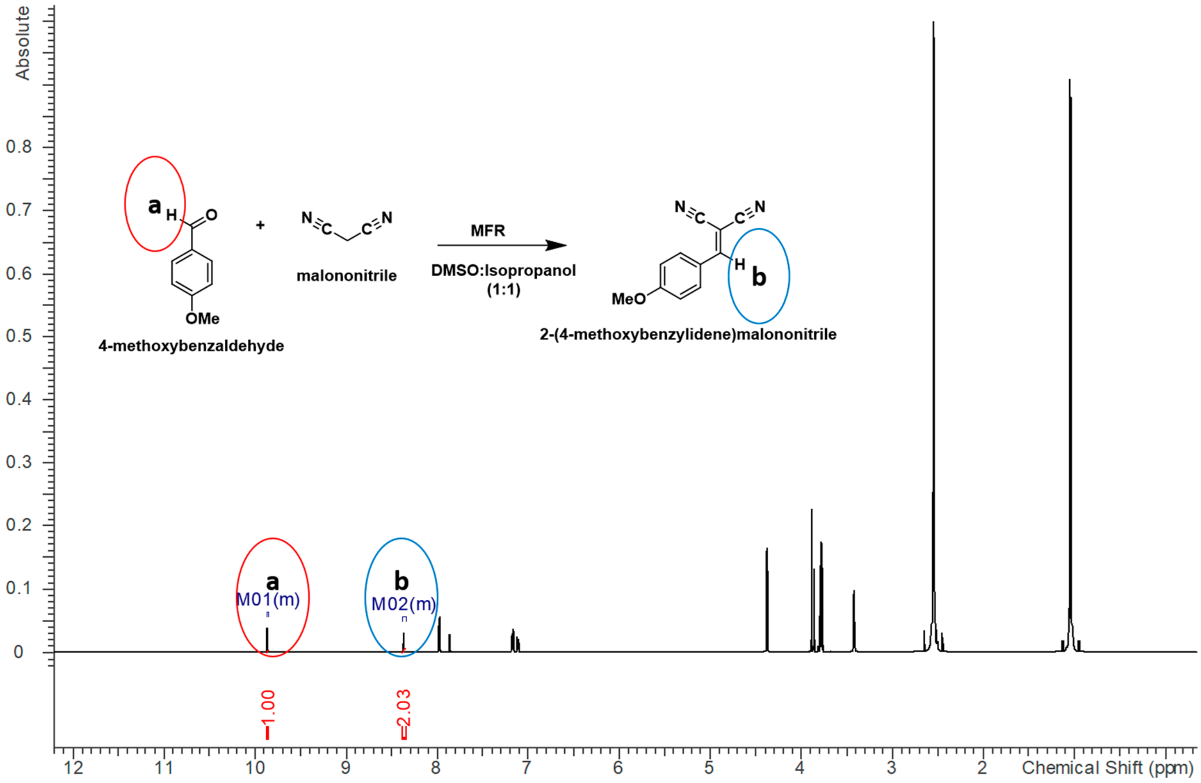

Figure S4: ^{1}H spectra for determination of conversion of the reaction between 4-methoxy benzaldehyde and malononitrile using polymeric gel composition C in solvent mixture, DMSO:IP (v:v=1:1) for 8 h.

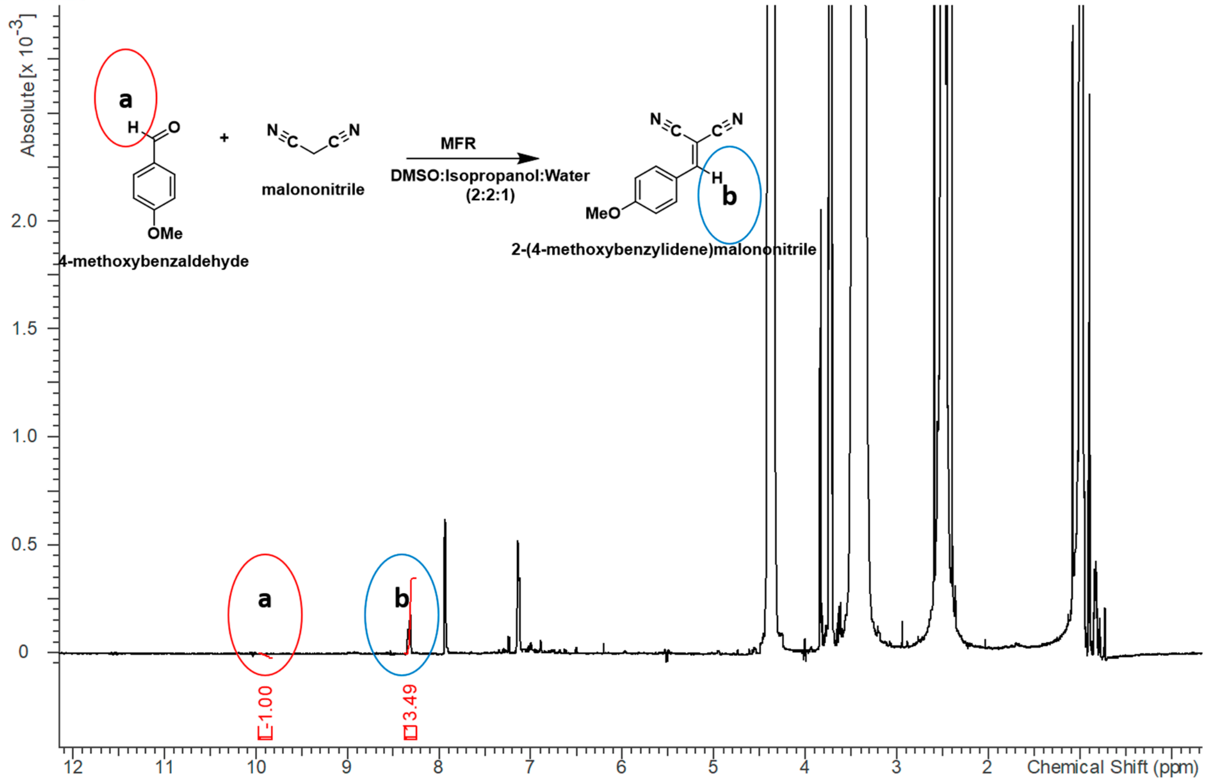

Figure S5: ^{1}H spectra for determination of conversion of the reaction between 4-methoxy benzaldehyde and malononitrile using polymeric gel composition A in solvent mixture, DMSO:IP:W (v:v:v=2:2:1) for 8 h.

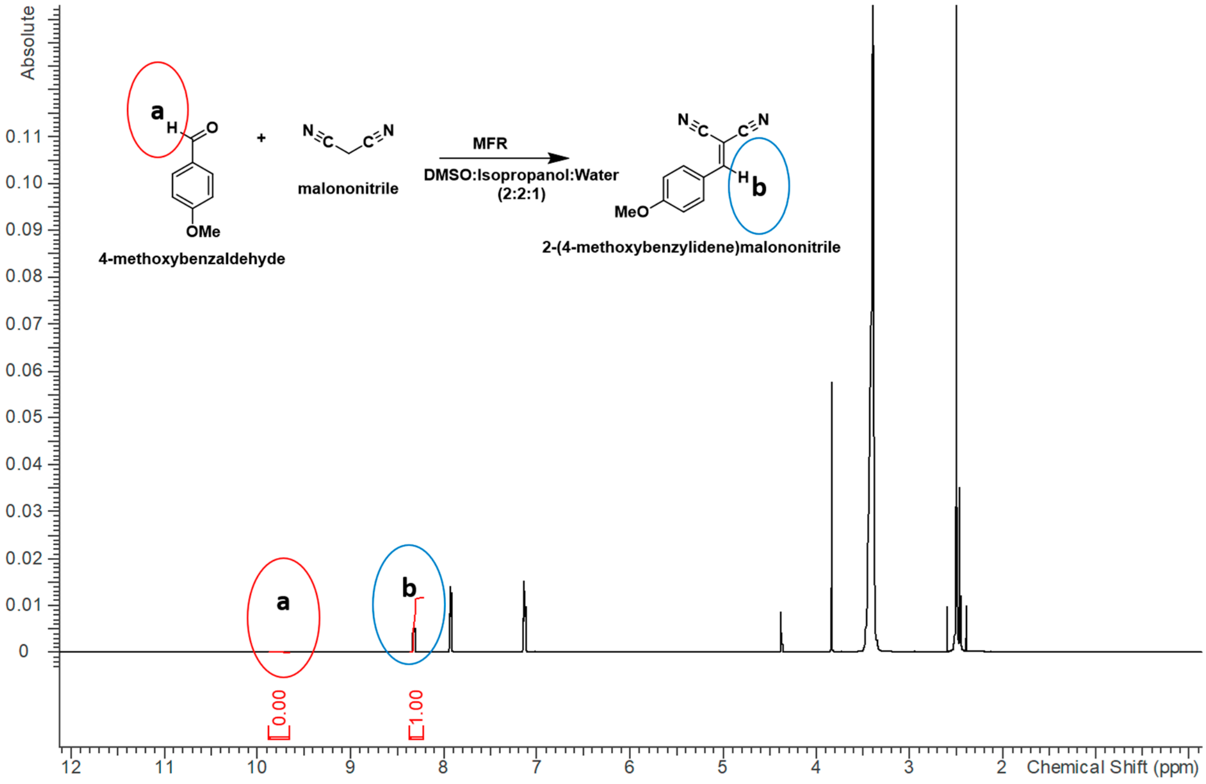

Figure S6: ^{1}H spectra for determination of conversion of the reaction between 4-methoxy benzaldehyde and malononitrile using polymeric gel composition B in solvent mixture, DMSO:IP:W (v:v:v=2:2:1) for 8 h.

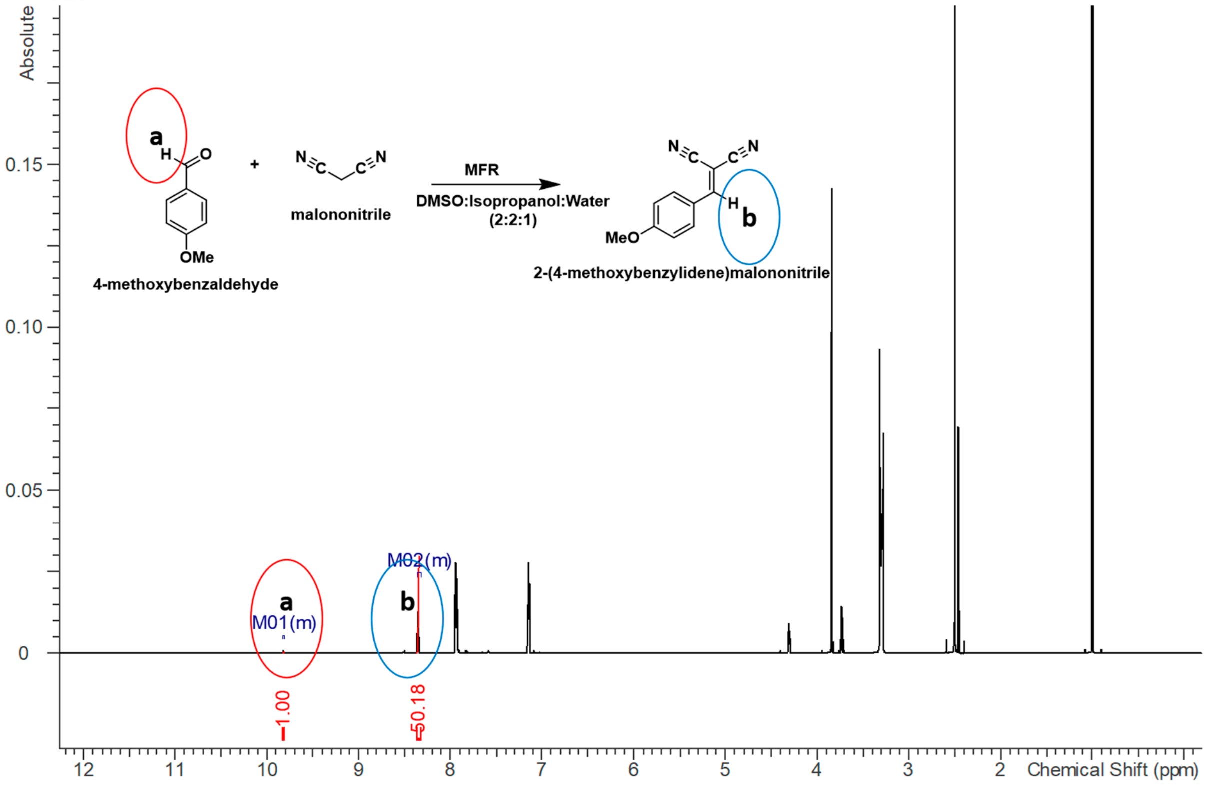

Figure S7: ^{1}H spectra for determination of conversion of the reaction between 4-methoxy benzaldehyde and malononitrile using polymeric gel composition C in solvent mixture, DMSO:IP:W (v:v:v=2:2:1) for 8 h.

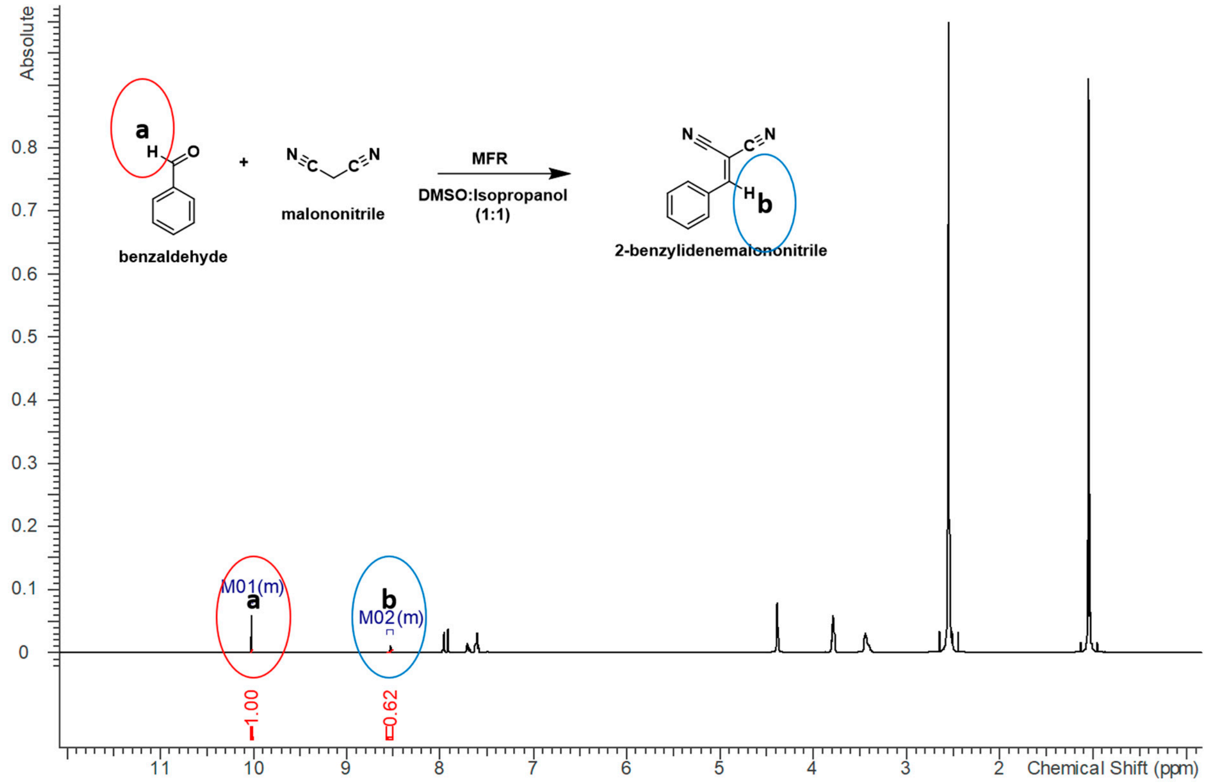

Figure S8: ^{1}H spectra for determination of conversion of the reaction between benzaldehyde and malononitrile using polymeric gel composition A in solvent mixture, DMSO:IP (v:v=1:1) for 8 h.

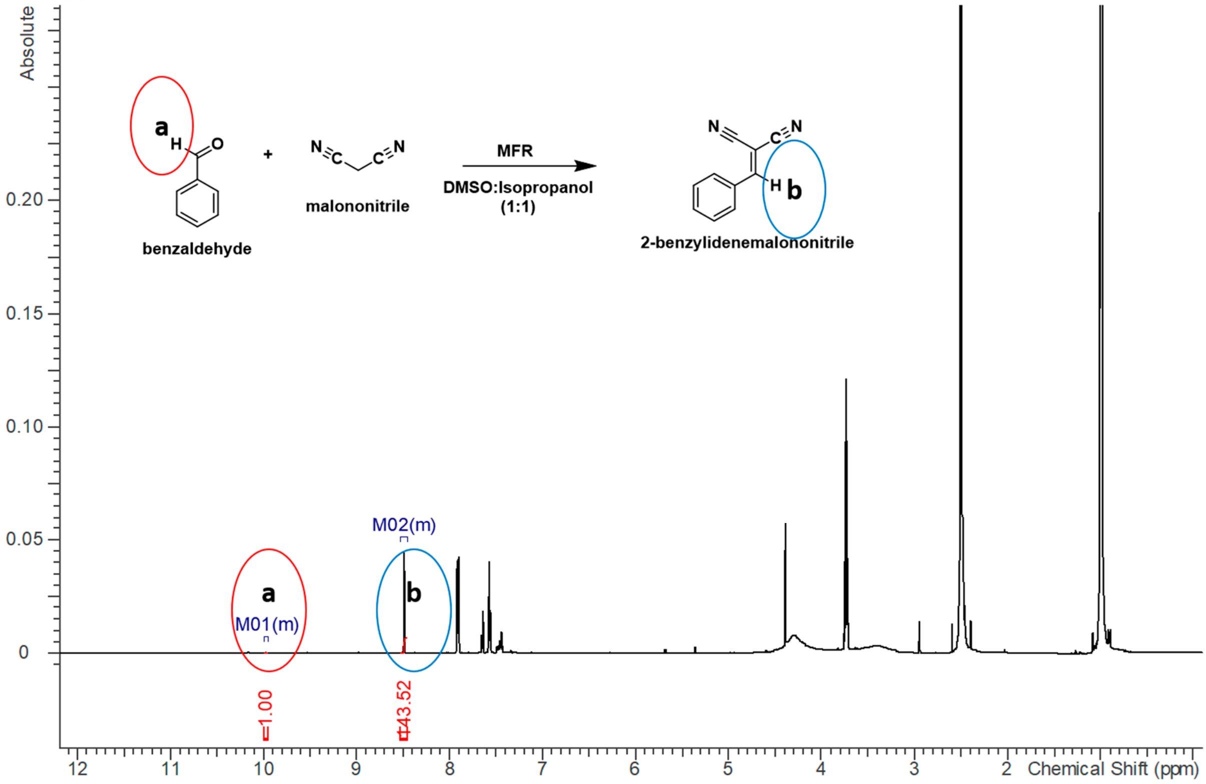

Figure S9: ^{1}H spectra for determination of conversion of the reaction between benzaldehyde and malononitrile using polymeric gel composition B in solvent mixture, DMSO:IP (v:v=1:1) for 8 h.

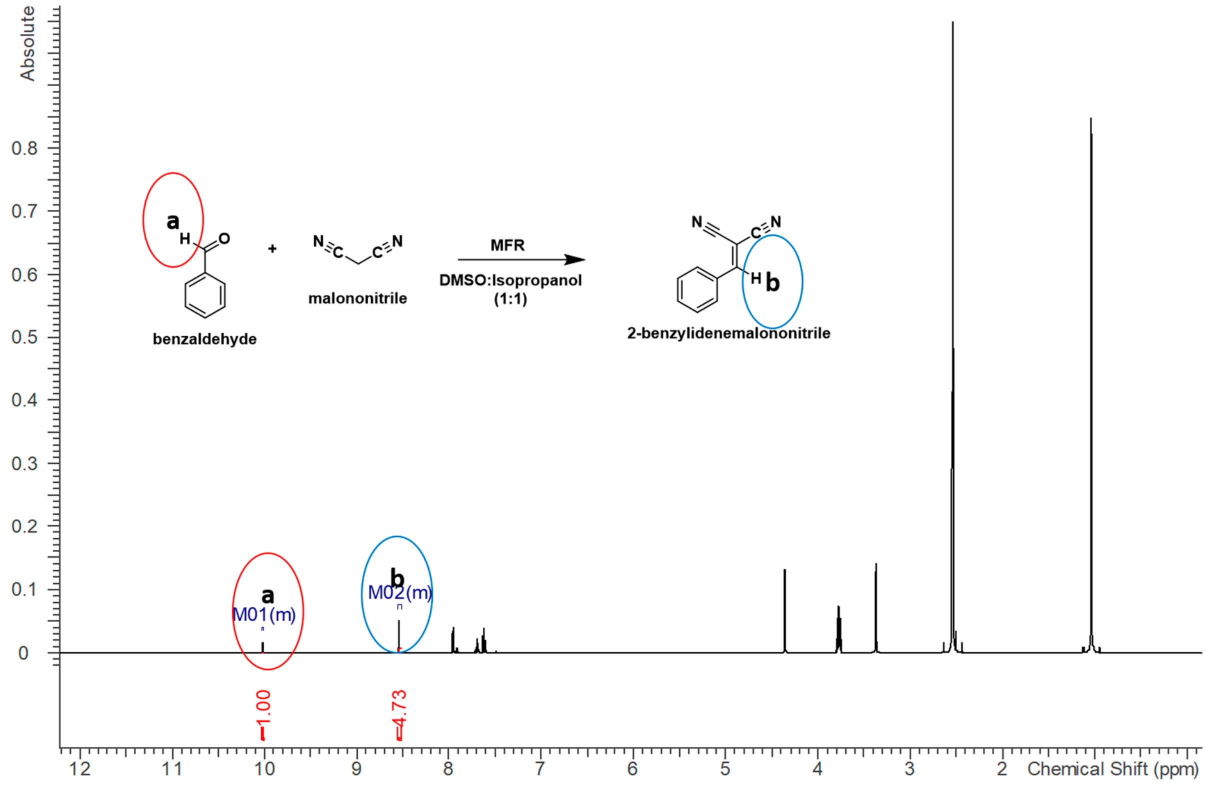

Figure S10: ^{1}H spectra for determination of conversion of the reaction between benzaldehyde and malononitrile using polymeric gel composition C in solvent mixture, DMSO:IP (v:v=1:1) for 8 h.

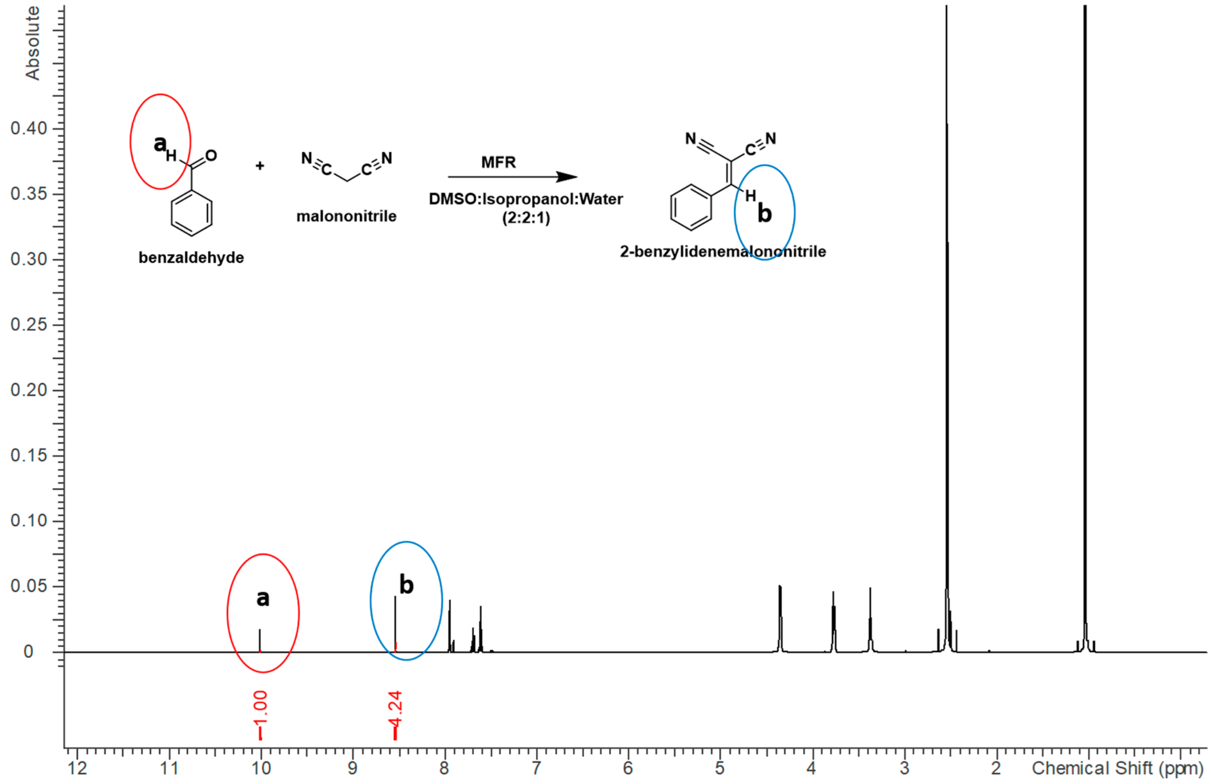

Figure S11: ^{1}H spectra for determination of conversion of the reaction between benzaldehyde and malononitrile using polymeric gel composition A in solvent mixture, DMSO:IP.W (v:v:v=2:2:1) for 8 h.

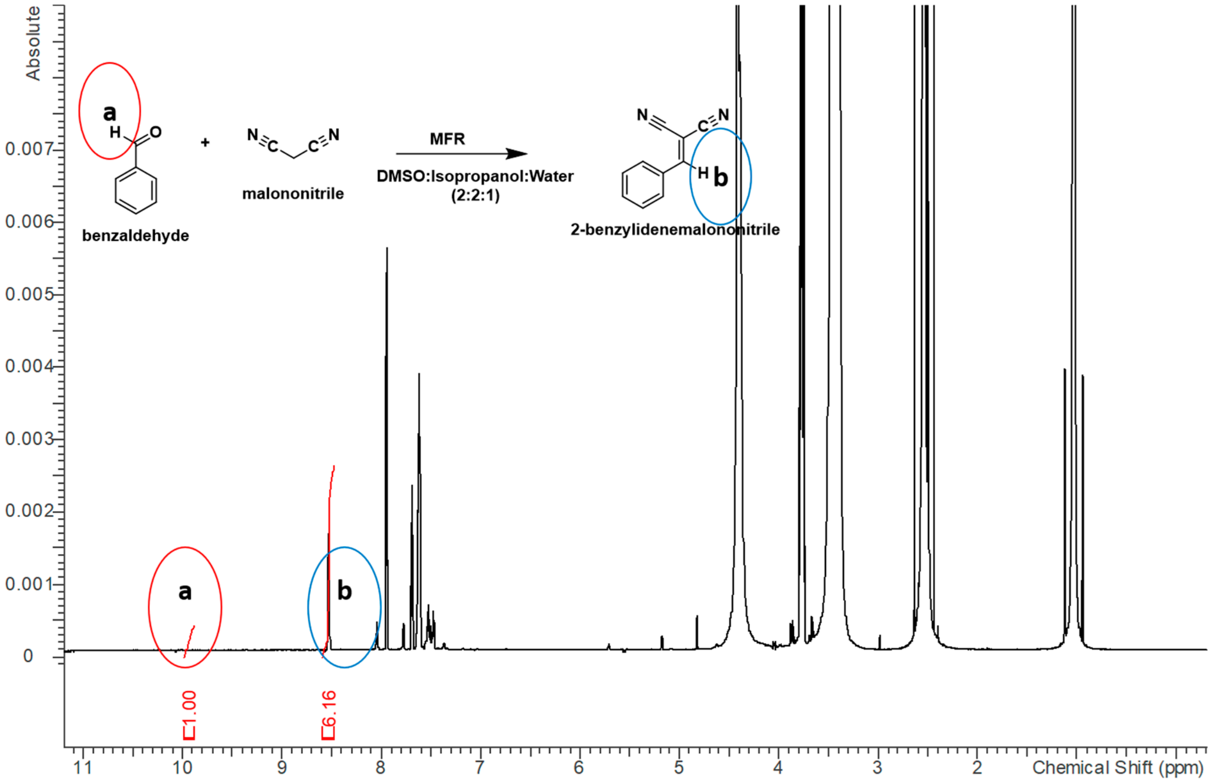

Figure S12: ^{1}H spectra for determination of Conversion of reaction between benzaldehyde and malononitrile using polymeric gel composition B in solvent mixture, DMSO:IP.W (v:v:v=2:2:1) for 8 h.

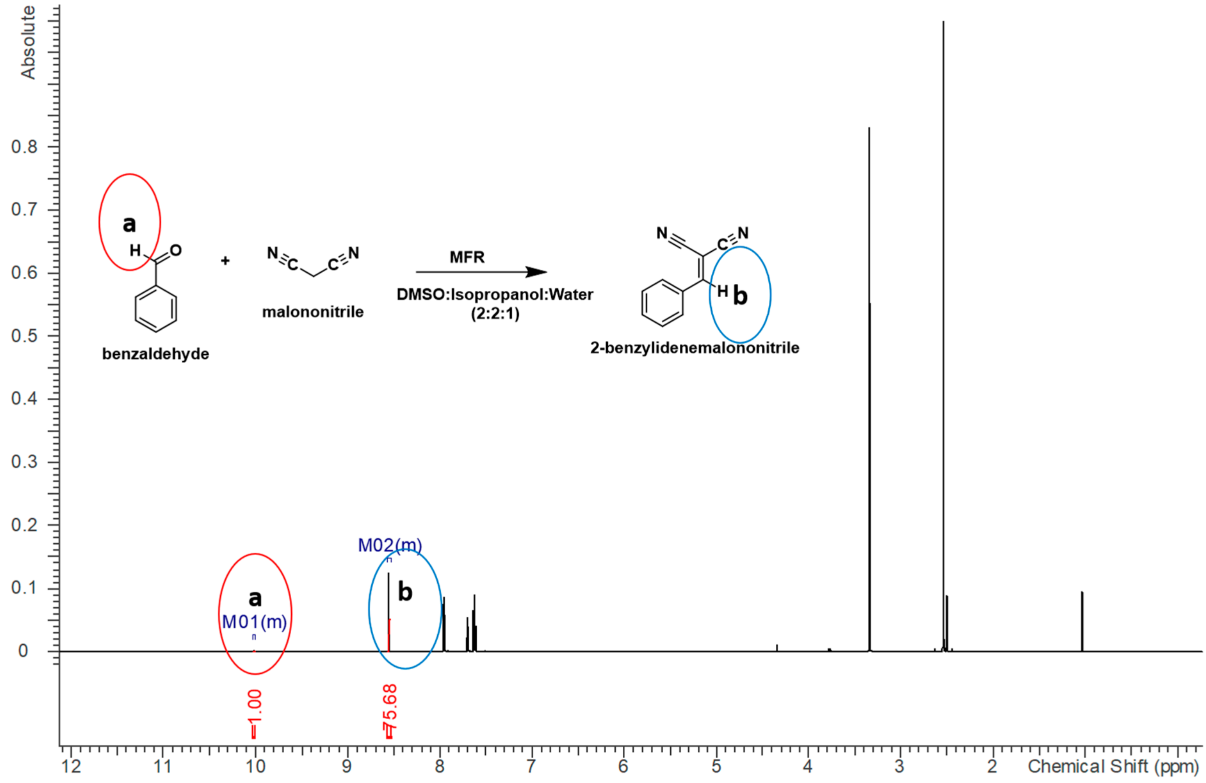

Figure S13: ^{1}H spectra for determination of Conversion of reaction between benzaldehyde and malononitrile using polymeric gel composition C in solvent mixture, DMSO:IP.W (v:v:v=2:2:1) for 8 h.

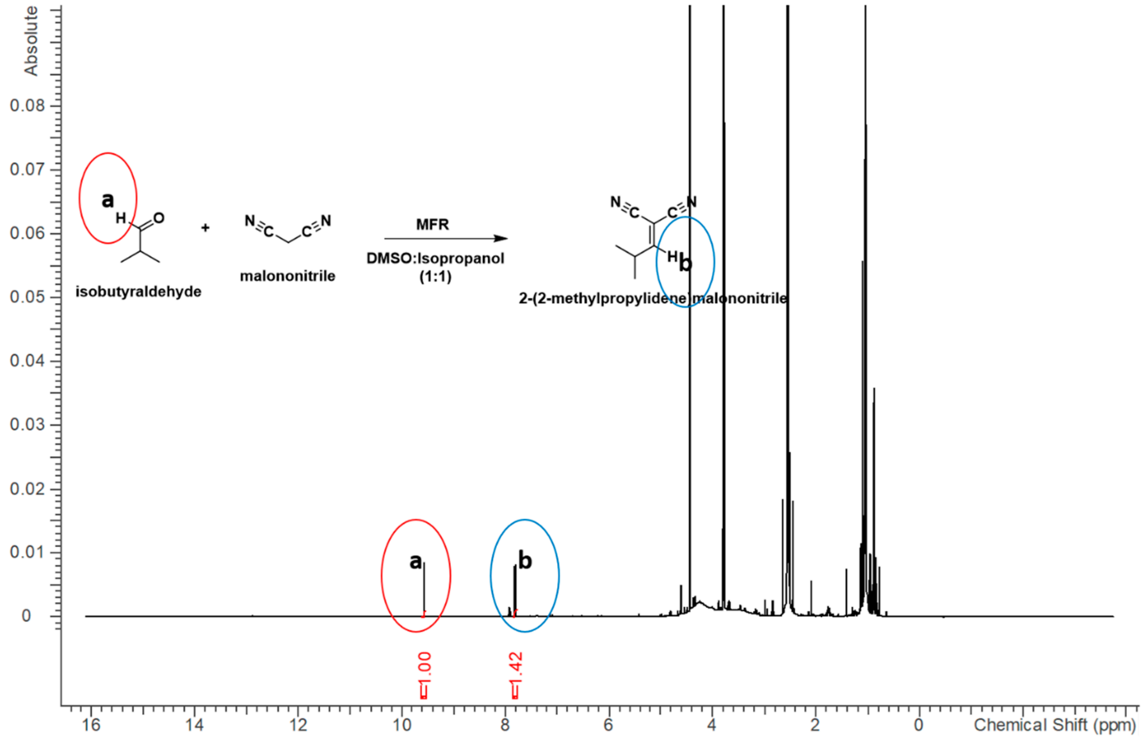

Figure S14: ^{1}H spectra for determination of conversion of reaction between iso-butyraldehyde and malononitrile using polymeric gel composition A in solvent mixture, DMSO:IP (v:v=1:1) for 8 h.

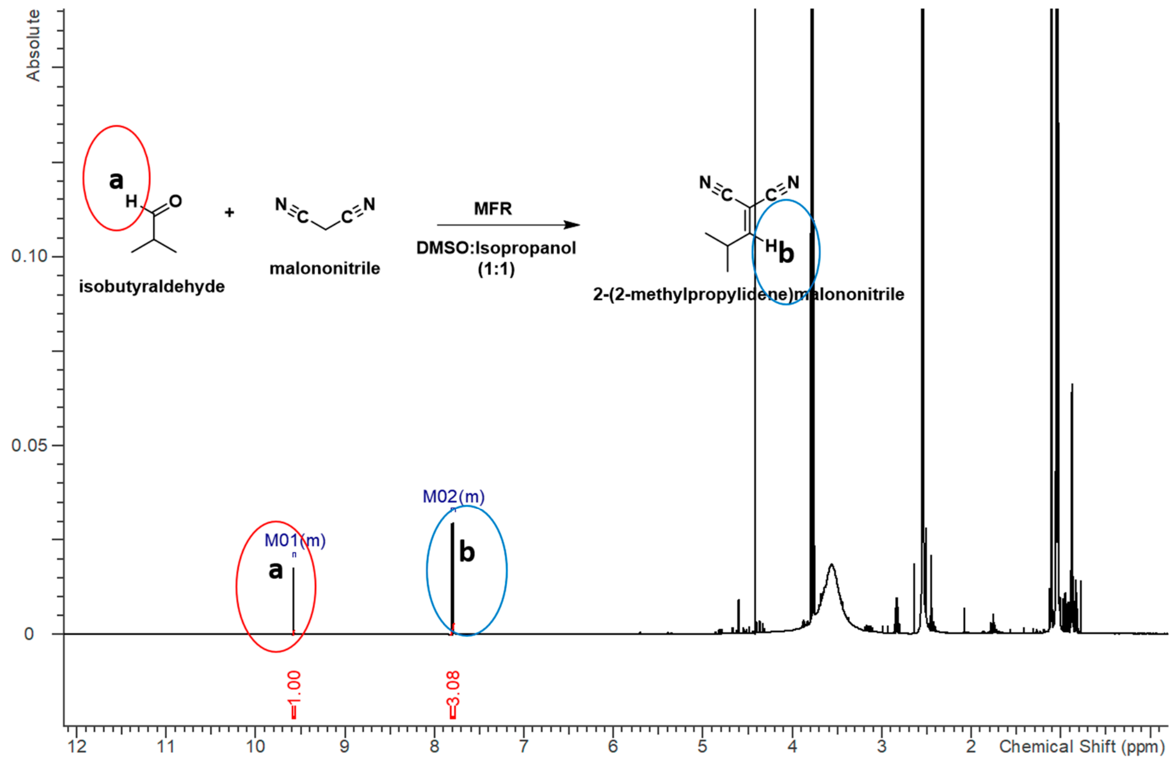

Figure S15: ^{1}H spectra for determination of conversion of reaction between iso-butyraldehyde and malononitrile using polymeric gel composition B in solvent mixture, DMSO:IP (v:v=1:1) for 8 h.

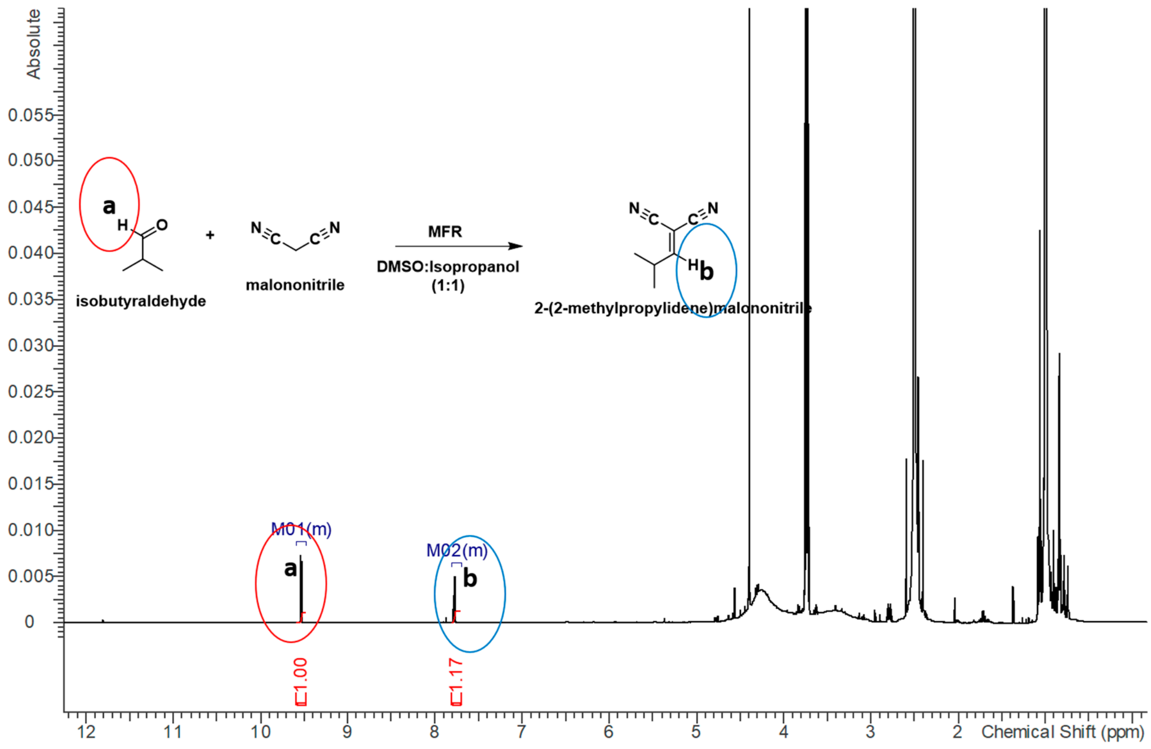

Figure S16: ^{1}H spectra for determination of conversion of reaction between iso-butyraldehyde and malononitrile using polymeric gel composition C in solvent mixture, DMSO:IP (v:v=1:1) for 8 h.

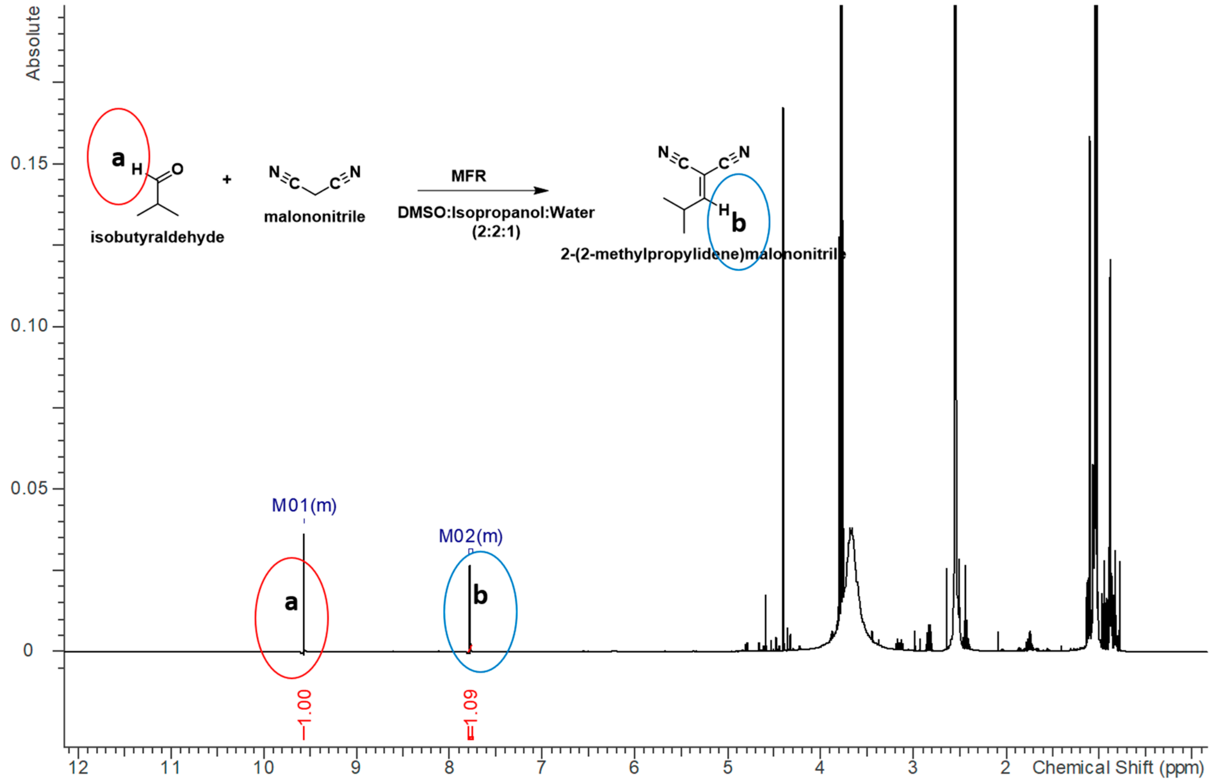

Figure S17: ^{1}H spectra for determination of conversion of reaction between iso-butyraldehyde and malononitrile using polymeric gel composition A in solvent mixture, DMSO:IP.W (v:v:v=2:2:1) for 8 h.

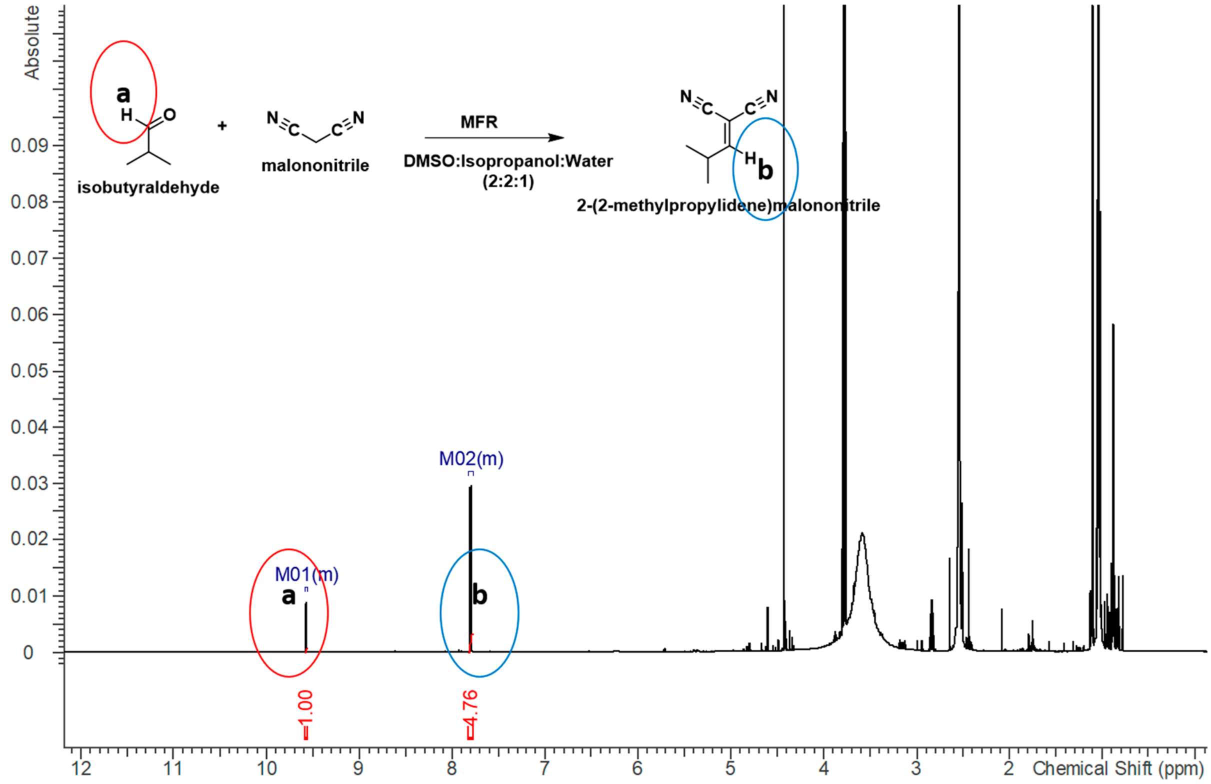

Figure S18: ^{1}H spectra for determination of conversion of reaction between iso-butyraldehyde and malononitrile using polymeric gel composition B in solvent mixture, DMSO:IP.W (v:v:v=2:2:1) for 8 h.

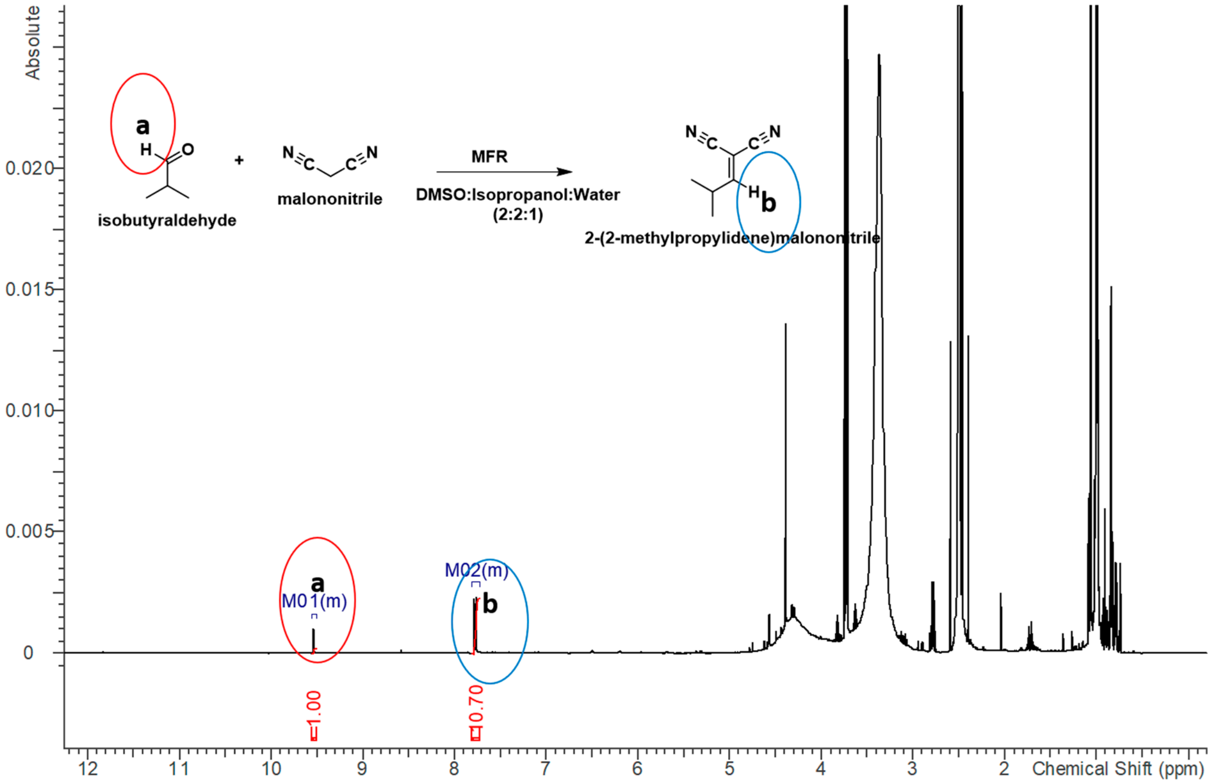

Figure S19: ^{1}H spectra for determination of conversion of reaction between iso-butyraldehyde and malononitrile using polymeric gel composition C in solvent mixture, DMSO:IP.W (v:v:v=2:2:1) for 8 h.

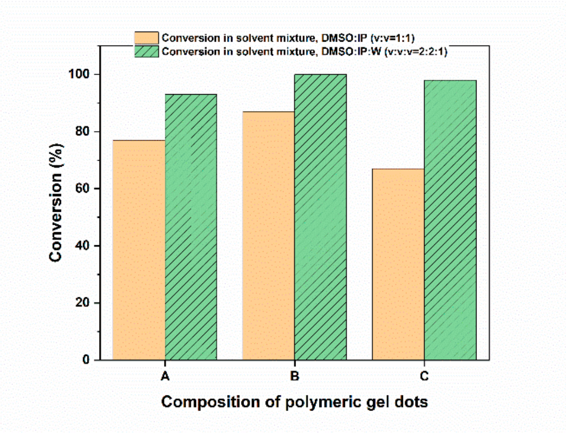

i

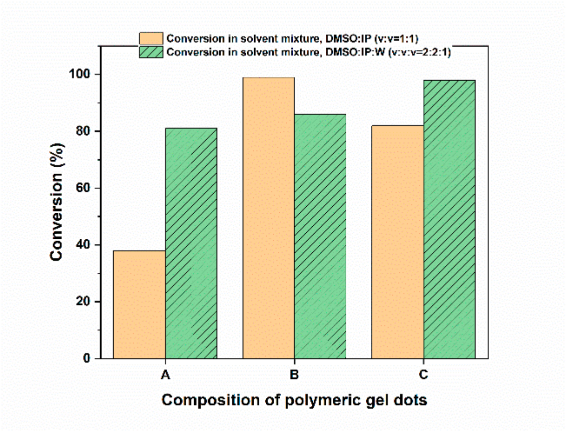

ii

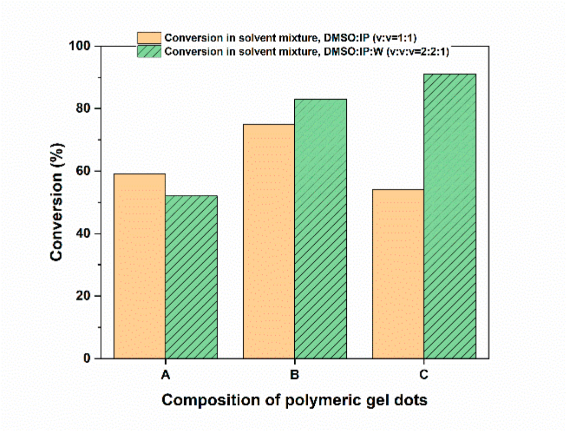

iii

Figure S20: Conversion of the reaction with malononitrile and different aldehydes i) 4-methoxybenzaldehyde, ii) benzaldehyde and iii) isobutyraldehyde using various polymeric gel dots compositions A, B and C for 8 h.
